# Properties of 2-locus genealogies and linkage disequilibrium in temporally structured samples

**DOI:** 10.1093/genetics/iyac038

**Published:** 2022-03-16

**Authors:** Arjun Biddanda, Matthias Steinrücken, John Novembre

**Affiliations:** Department of Human Genetics, University of Chicago, Chicago, IL 60637, USA; Department of Human Genetics, University of Chicago, Chicago, IL 60637, USA; Department of Ecology and Evolution, University of Chicago, Chicago, IL 60637, USA; Department of Human Genetics, University of Chicago, Chicago, IL 60637, USA; Department of Ecology and Evolution, University of Chicago, Chicago, IL 60637, USA

**Keywords:** linkage disequilibrium, ancient DNA, population genetics

## Abstract

Archeogenetics has been revolutionary, revealing insights into demographic history and recent positive selection. However, most studies to date have ignored the nonrandom association of genetic variants at different loci (i.e. linkage disequilibrium). This may be in part because basic properties of linkage disequilibrium in samples from different times are still not well understood. Here, we derive several results for summary statistics of haplotypic variation under a model with time-stratified sampling: (1) The correlation between the number of pairwise differences observed between time-staggered samples ($\pi_{\Delta t}$) in models with and without strict population continuity; (2) The product of the linkage disequilibrium coefficient, *D*, between ancient and modern samples, which is a measure of haplotypic similarity between modern and ancient samples; and (3) The expected switch rate in the Li and Stephens haplotype copying model. The latter has implications for genotype imputation and phasing in ancient samples with modern reference panels. Overall, these results provide a characterization of how haplotype patterns are affected by sample age, recombination rates, and population sizes. We expect these results will help guide the interpretation and analysis of haplotype data from ancient and modern samples.

## Introduction

Multilocus properties of genetic variation have been useful for studying evolutionary processes and maximizing the information extracted from population genetic data. Patterns of multilocus variation are shaped by mutation and recombination events, generating novel combinations of alleles on chromosomes (i.e. haplotypes). The nonrandom association of alleles between 2 (or more) loci is known as linkage disequilibrium (LD; Lewontin and Kojima 1960; Hill and Robertson 1968; Slatkin 2008). Common measures of LD include the covariance and correlation in allelic state at 2 loci on the same haplotype within a sample (*D* and *r*^2^, respectively; Hill and Robertson 1968; Slatkin 2008). The decay of LD as a function of the distance between genetic variants plays an important role in dating evolutionary events (e.g. Moorjani *et al.* 2016), determining the accuracy of complex trait prediction (e.g. Vilhjálmsson *et al.* 2015), and moderating the power to map trait-associated loci (e.g. Wray 2005; Spencer *et al.* 2009).

One approach for modeling variation at multiple loci has been through the use of coalescent theory (Kingman 1982; Hudson 1985). The coalescent process at multiple loci can involve both recombination (splitting events) and coalescence (joining events) of ancestral lineages, which means that there can be a different number of lineages ancestral to a sample at each locus at a given point in time (Hudson 1985; Simonsen and Churchill 1997; Durrett 2008). Based on a 2-locus coalescent model, Hudson (2001) developed a composite likelihood approach to estimate fine-scale recombination rates in early sequencing datasets. This initial approach paved the way for subsequent methods to estimate fine-scale recombination rates in humans, accommodating increasing model complexity (McVean *et al.* 2004; Auton and McVean 2007; Kamm *et al.* 2016; Spence and Song 2019). Also using a 2-locus coalescent model, McVean (2002) was able to express metrics of LD in terms of properties of coalescent times. As the impact of changing demographic history on coalescent times is relatively straightforward, this advance enabled a more intuitive understanding of the impact of demographic history and sampling design on expected patterns of LD in data (McVean 2002; Wakeley and Lessard 2003).

A second major modeling framework for LD has been via “haplotype copying” models, such as the Li and Stephens’ model (Li and Stephens 2003; Song 2016). Haplotype copying models provide a computationally efficient approximation for the likelihood of observed haplotype data generated with recombination (Fearnhead and Donnelly 2001). As a result, they have become a backbone of many analyses of population-genomic data, such as genotype imputation (e.g. Howie *et al.* 2009), haplotype phasing (e.g. Loh *et al.* 2016), and local ancestry inference (Price *et al.* 2009; Lawson *et al.* 2012).

In an increasing number of settings, samples are not all from the same time point. This is exemplified by the growing study of archeogenetics, also known as ancient DNA (aDNA) studies (reviewed in Slatkin and Racimo 2016; Llamas *et al.* 2017; Skoglund and Mathieson 2018). Archeogenetic studies of humans have been able to reliably obtain genetic data from samples up to 45,000 years before present, although the majority of samples are from the past ∼15,000 years (Skoglund and Mathieson 2018).

For single-locus data, genealogical models have been developed to quantify the impact of ancient samples on population genetic statistics, such as the expected site-frequency spectrum, the number of variants private to an ancient sample, and *F_ST_* (Rodrigo and Felsenstein 1999; Forsberg *et al.* 2005; Ortega-Del Vecchyo and Slatkin 2018). In contrast, the impact of time-separation on patterns of LD has not been fully explored.

Here, we characterize patterns of haplotype variation in temporally stratified samples using a genealogical perspective. Analogous approaches for time-stratified samples in a coalescent framework have generally not been developed for the case of 2 or more recombining loci. One exception is the approach of Dialdestoro *et al.* (2016) that uses importance sampling over the space of latent ancestral recombination graphs when calculating the likelihood of observed sequence data for haplotypes at multiple time-points. Our work here contrasts to that of Dialdestoro *et al.* (2016) in that we obtain analytic solutions for 2-locus scenarios and for the haplotype copying model. The work presented here is complementary to previous work by Terhorst *et al.* (2015) who modeled how allele frequencies change for multiple loci using a Gaussian approximation to the Wright–Fisher model, though here we approach the problem from a coalescent perspective.

We primarily consider statistics based on 2 haplotypes as a starting point for representing the impact of time-stratified sampling across multiple loci. However, we also explore the statistic $\sigma_{t}^{2}$, whose properties can be understood as an expectation over 4 haplotypic states. We focus on these simplified scenarios as they are analytically tractable, while still providing insight on expected patterns in data (Hudson 1985; McVean 2002). We first show how time-stratified sampling affects the joint properties of genealogies at 2 loci, demonstrating that the time gap between a pair of samples has an impact on the rate of decay in the correlation of genealogical statistics and corresponding patterns of variation with recombination distance. We also analyze the behavior of fitting the haplotype copying model with samples of different ages, in particular when the test haplotype is from a time-point in the past compared with a modern haplotype panel. Overall, our results show the effect of time-stratified sampling on expected patterns of haplotypic variation, and their implications for the further development of population genetic methods.

## Methods

### Coalescent simulations and calculation of pairwise-differences

We used *msprime* (Kelleher *et al.* 2016) to perform all coalescent simulations used throughout the article. For simulations of 2 loci, we used a customized recombination map to reflect 2 nonrecombining loci of a given size separated by a specified absolute recombination rate. For the simulations of haplotypes, we use the default simulation method and a uniform recombination map (default $r=10^{-8}$ per-basepair per-generation). To calculate a pairwise-coalescent effective *N_e_* to compare our constant-population-size theory for 2 loci with simulations under varying demographic history, we took a Monte-Carlo approach using 10^4^ coalescent simulations to compute the mean marginal pairwise coalescent time $\bar{T_{2}}$ from simulations and compute ${\hat{N}}_{e}$ as $2\bar{T_{2}}$.

#### Monte-Carlo simulation of correlation in pairwise differences

To verify our comparisons of the theoretical prediction of $\mathrm{Corr}(\pi_{A},\pi_{B})$ with data, we simulated 2 loci as described above with a mutation rate θ=0.4 (approximately equivalent to a 1-kb window with human scale parameters) for 100 log-spaced points from $\rho \in [10^{-4},10^{2}]$. When estimating $\mathrm{Corr}(\pi_{A},\pi_{B})$, we conducted 100,000 independent simulations and estimated the Pearson correlation using the pearsonr function in the scipy package (Virtanen *et al.* 2020). The standard error of the correlation was calculated using the asymptotic formula: $\left({\hat{s}}_{r}=\sqrt{\frac{1-{\hat{r}}^{2}}{n-2}}\right)$.

For estimating the correlation in pairwise differences, we simulated 20 replicates of 20 Mb haplotypes and calculated a Monte-Carlo estimator of the mean correlation in segregating sites at different recombination distances. The estimation proceeds as follows: (1) we split the chromosome into nonoverlapping windows of length *L* basepairs (default: 1 kb); (2) for each of 5,000 Monte-Carlo samples we choose a window *S_A_* and define a paired window a recombination distance *r* from it (randomly choosing the direction to search); (3) compute the empirical Pearson correlation coefficient of the number of pairwise differences $\mathrm{Corr}(\pi_{A},\pi_{B})$ across the 5,000 paired windows. Standard errors were computed using the asymptotic formula above, using the 20 replicate chromosomes. For estimation with the real whole-genome sequencing data, we use 30 log-spaced bins over the range $r=(10^{-5},10^{-3})$, where *r* is in Morgans to calculate Monte-Carlo estimates of the correlation in pairwise differences. Unless otherwise specified in the text, error bars reflect 2 standard errors from the mean. When translating from years to generations for comparison of models to our theoretical predictions, we use a generation time of 30 years per generation from Fenner (2005).

#### Monte-Carlo estimation of joint LD

To estimate the product of LD across timepoints (Equation 7), we used Monte-Carlo simulations of 500 modern and ancient haplotypes in a model of constant population size of $N_{e}=10^{4}$. We conducted 10 replicate simulations of 1 megabase haplotypes with the mutation rate and recombination rate set to $10^{-8}$ per basepair per generation. We applied a filter of the minor allele frequency pooled across timepoints at >5% when calculating the joint LD coefficient. We additionally bin by genetic distance using the automatic histogram binning in *scipy* (Virtanen *et al.* 2020). For very low values of *ρ*, there are too few mutations co-occurring at such short distances in our simulations so we set a lower-bound of *ρ *= 1 when plotting Fig. 5.

### Analysis of ancient whole-genome sequencing data

For our analysis of whole-genome aDNA data, we compared single nucleotide variants observed in the *LBK* and *Ust-Ishim* samples (Lazaridis *et al.* 2014; Fu *et al.* 2014). Variants were called using *samtools mpileup -C50* and were subsequently filtered using the same criterion as in de Barros Damgaard *et al.* (2018).

To account for not having resolved haplotypes in the ancient samples, we scale the observed differences by the probability that they would be observed in a haplotype randomly sampled from the diploid genome (e.g. 0.5 if heterozygote in ancient sample, 1 if opposing homozygote in the ancient sample). For modern samples, we used haplotypes from the 1000 Genomes Project Phase 3 Dataset (Auton *et al.* 2015).

We computed the correlation in pairwise differences in nonoverlapping 1-kb windows and applied a mappability mask to account for varying coverage in the modern sample by normalizing (Auton *et al.* 2015). Standard errors were estimated using a nonparametric bootstrap across 22 autosomes. To compare 2 empirical curves of $\hat{\mathrm{Corr}}(\pi_{A},\pi_{B})$, we apply a 2-sided Binomial sign test to test the proportion of recombination distance bins for which 1 ancient sample has a higher correlation and test against the null hypothesis that the proportion is 0.5.

### Parameter estimation in the haplotype copying model

We implemented a version of the haplotype copying model proposed by Lawson *et al.* (2012) that accounts for the genetic map distances between subsequent single-nucleotide polymorphisms. The Hidden Markov Model (HMM) is defined as follows. The transition probabilities between hidden states, *X_l_*, where *X_l_* represents the haplotype in the panel that the test haplotype copies off of at site *l*:

(1) $$P(X_{l}=x\prime |X_{l-1}=x)=\{\begin{array}{cc} e^{-\lambda g_{l}}+\frac{1}{K}(1-e^{-\lambda g_{l}}), & x\prime =x\\ \frac{1}{K}(1-e^{-\lambda g_{l}}), & \text{else} \end{array},$$

where *g_l_* is the *genetic distance* between markers *l−*1 and *l* (in Morgan), *K* is the size of the haplotype reference panel, and *λ* is the “jump rate” or rate at which the model transitions between the haplotype copying states.

The emission probabilities can be similarly characterized, using a parameter *ϵ* that represents the probability of a copying error:

(2) $$P(h_{l}=a\prime |X_{l}=a)=\{\begin{array}{cc} \epsilon , & a\prime \neq a\\ (1-\epsilon ), & a\prime =a \end{array},$$

where *h_l_* is the allelic state of the query haplotype at site *l*.

We use 2D numerical optimization from *scipy.optimize* (Virtanen *et al.* 2020) to jointly estimate the maximum-likelihood estimates $\hat{\lambda }$ and $\hat{\epsilon }$. Unless specifically stated, we use the joint parameter estimates in our results for both simulated and empirical data. For profile maximum-likelihood estimates of $\hat{\lambda }$, we use Brent optimization within the range [$0,\ldots ,10^{6}$] with a fixed $\epsilon =10^{-2}$. We estimate standard errors for $\hat{\lambda }$ and $\hat{\epsilon }$ using a finite-difference approximation to the second derivative of the joint log-likelihood surface.

All simulations under the haplotype copying model were conducted using chromosomes of 40 megabases, and recombination and mutation rates of $10^{-8}$ per basepair per generation. Every modern panel consisted of *K *=* *100 haplotypes (unless otherwise specified). We also ascertained to variants with a minor allele frequency >5% in the modern panel.

### Analysis of male X-chromosomes in 1,240K human aDNA dataset

The human aDNA data that we used for our analysis of the haplotype copying model (see *Online Resources*) are typed at a set of 1,233,013 sites across the genome and downloaded from the David Reich Laboratory’s website. Genotypes are drawn using psuedohaploid sampling based on the available reads at these sites. We filtered the data based on the following criteria for our analysis while restricting to the X chromosome: (1) Must be a male sample; (2) Samples must not have a significant amount of modern DNA contamination (e.g. “PASS” contamination checks); and (3) Samples must have ≥8,000 nonmissing variants across the X chromosome. Following this filter, the median autosomal coverage for the remaining samples is 2.303×, and an average of 1.29 sites per 25 kb on the X-chromosome.

Following these filters, we have a total set of 798 samples for which we estimated the maximum-likelihood jump rate under the haplotype copying model. To minimize confounding via spatial variables, we chose a centroid location (48°N latitude, 6°E longitude) and only retained samples within 1,500 km of this centroid. Following this filtering step, there are 344 samples that are used for the main figures (Fig. 7).

We performed estimation of the haplotype copying jump rate across all of the 798 originally filtered samples using 3 different haplotype reference panels (49 CEU haplotypes [“CEU”]; 240 EUR haplotypes [“EUR”]; 1,233 haplotypes [“FULLKG”]) for the X-chromsome from the 1000 Genomes Phase 3 dataset (Auton *et al.* 2015). In all cases, we used the sex-averaged recombination map for the X-chromosome from Kong *et al.* (2010). For linear modeling of the jump-rate as a function of the sample age, we used the *OLS* function of *statsmodels* package (Seabold and Perktold 2010). When comparing the real data against simulations under the demographic models inferred by Tennessen *et al.* (2012) and Browning and Browning (2015), we use *n *=* *49 modern day CEU haplotypes and sampled haplotypes at ages corresponding to the real data using a generation time of 30 years per generation (Fenner 2005). We additionally scaled each demographic model by 3/4 to reflect the reduced effective size of the X-chromosome.

## Results

### Two-locus genealogical properties

**Fig. A1.**
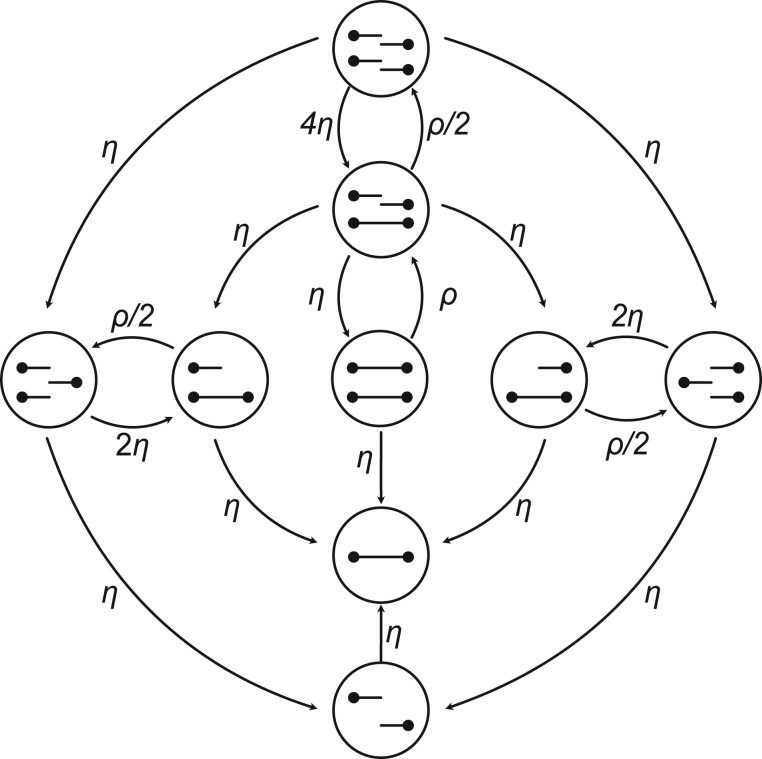
Markov chain model for the ancestral process at 2 loci from Simonsen and Churchill (1997). In all settings for 2 modern haplotypes, we assume that we start from the state in the middle (state “0”) in all applications, which means that all sampled haplotypes are coupled. The parameter *η* represents the coalescent rate and the parameter *ρ* represents the recombination rate (measured in coalescent units). Figure adapted from Hobolth and Jensen (2014).

To model 2 haplotypes at 2 loci with time-stratified sampling, we adapted a previously developed continuous time Markov process for modeling ancestral lineages at 2 loci (Hudson 1983, 1990; Simonsen and Churchill 1997). The states in the model are triplets [e.g. (2, 0, 0)] that depict the number of lineages ancestral to both loci, locus 1, or locus 2, respectively. Coalescence and recombination events eventually lead to an absorbing state where *both* haplotypes have coalesced at *both* loci [the state (1, 0, 0), Fig. A1]. Analytical results for joint moments in the coalescent times in this model have been previously obtained for the case where samples are taken at the present (Hudson 1983; Simonsen and Churchill 1997; Durrett 2008, Chapter 3).

**Fig. 1.**
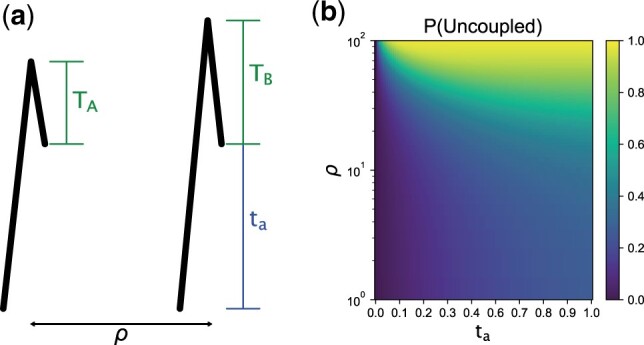
a) Schematic of genealogies at 2 loci separated by a population-scaled recombination distance *ρ* ($\rho =4N_{e}r$). The parameter *t_a_* represents the sampling time of the haplotype (measured in coalescent units, i.e. scaled by $2N_{e}$). The random variables *T_A_* and *T_B_* are the additional time to coalescence at locus *A* & *B*, after *t_a_*. b) The probability of the modern haplotype being “uncoupled” at the time of ancient sampling as a function of *t_a_* and *ρ*. In this setting, “uncoupled” means that the ancestral lineages at locus *A* and *B* are not on the same haplotype, enhancing the probability of different *T_A_* and *T_B_* occurring at each locus.

Here, to analyze the case of time-stratified sampling, we assume that one of the haplotypes has been sampled at time *t_a_* in the past (in coalescent units) and the other at the present. With this time gap in sampling, there are 2 natural phases in the ancestral process: (1) the time between the present and when the ancient haplotype is sampled ($t<t_{a}$), and thus only the lineage of the modern haplotype can evolve at each locus, and (2) the time when the lineages of both haplotypes (modern and ancient) are evolving through the full state space of the ancestral process ($t\geq t_{a}$).

For this 2-phase ancestral process, we derived expressions for the covariance between the *T_MRCA_’s* at 2 loci (*A* and *B*), as well as the total branch lengths (*L_A_*, *L_B_*) separated by a population-scaled recombination distance, $\rho =4N_{e}r$, where *r* is the per-generation probability of recombination.

The derivation proceeds by recognizing that a key aspect of the 2-phase process is the effect of recombination during the first phase, when only the modern lineage is evolving backwards in time ($t<t_{a}$, see Appendix A). During this phase the process has only 2 states, “uncoupled” and “coupled.” By “uncoupled,” we mean that the ancestral lineages are evolving independently at each locus, whereas “coupled” means that they are evolving as a joint ancestral lineage. The starting state for the second phase of the ancestral process (when $t\geq t_{a}$) is either that the modern haplotype’s ancestral lineages are coupled at both loci or uncoupled from one another. We obtain the time-dependent probability of being in the uncoupled state by exponentiating the 2 × 2 rate matrix **Q** for the reduced state-space of the ancestral process during $t<t_{a},\;{\left(e^{Qt_{a}}\right)}_{0,1}$, where $Q=\left[\left[-\frac{\rho }{2},\frac{\rho }{2}],[1,-1\right]\right]$. By doing so and taking different limits, we find:

(3) $$\begin{array}{c} P_{t_{a}}(\text{uncoupled})=\frac{\rho (1-e^{-t_{a}(\frac{\rho }{2}+1)})}{\rho +2}\\ \approx \{\begin{array}{cc} \frac{t_{a}\rho }{2} & ,t_{a}\rho \ll 1,\\ \frac{\rho }{\rho +2} & ,t_{a}\to \infty ,\\ 1-e^{-t_{a}/2} & ,\rho \to \infty . \end{array} \end{array}$$

Figure 1b shows for either large time-separation (*t_a_*) or large population-scaled-recombination rates (*ρ*), it becomes more likely that the modern haplotype is in the uncoupled state by the time the process encounters the ancient haplotype. Since the remaining dynamics are the same as the 2-locus ancestral process with 2 contemporaneously sampled haplotypes, we thereafter leverage known results for the 2-locus ancestral process (Simonsen and Churchill 1997; McVean 2002; Durrett 2008, Chapter 3). In the next 2 sections, we take this modeling approach to derive the expectations of observable quantities from time-staggered haplotype data.

### Correlation in pairwise differences

The number of pairwise differences between 2 haplotypes at each of 2 loci is an observable summary of genetic variation at linked loci in time-sampled sequence data. To investigate the properties of the joint distribution on pairwise differences at 2 loci (locus *A* and *B*), we continue to assume a model with recombination occurring at a rate *ρ* between them and no recombination occurring within each. For each locus, as is typical in coalescent models, we assume an infinite-sites model with mutations arising on each lineage as a Poisson process with rate $\frac{\theta }{2}$, where $\theta =4N_{e}\mu L$, *μ* is the per-basepair per-generation mutation rate, *L* is the size of the locus (in basepairs), and *N_e_* is the effective population size.

Following the approach described in the preceding section, we derive the correlation of pairwise differences for the case with time-stratified sampling (see Appendix A). In particular, we use the fact that the correlation in the number of pairwise differences at locus *A* and *B* can be expressed in terms of the correlation in the total branch length between the loci (Wakeley and Lessard 2003; Hobolth *et al.* 2019). We find the correlation in pairwise differences between 2 loci to be:

(4) $$\mathrm{Corr}(\pi_{A},\pi_{B})=\frac{1}{1+\frac{2+t_{a}}{2\theta }}C\mathrm{orr}(L_{A},L_{B}),$$

where $\mathrm{Corr}(L_{A},L_{B})$ is the correlation in total branch length at locus *A* and locus *B*. In Appendix A (building on previous results from Hudson 1983; Simonsen and Churchill 1997; Durrett 2008, Chapter 3), we derive its exact form and several limiting values to be:

(5) $$\begin{array}{c} \mathrm{Corr}(L_{A},L_{B})=\frac{\rho +18}{\rho^{2}+13\rho +18}\\ \;\;-(1-e^{-t_{a}(\frac{\rho }{2}+1)})\left(\frac{\rho }{\rho +2}\right)\frac{\rho +12}{\rho^{2}+13\rho +18}\\ \approx \{\begin{array}{cc} \frac{\rho +18}{\rho^{2}+13\rho +18} & ,t_{a}\to 0,\\ \frac{\rho +18}{\rho^{2}+13\rho +18}-\frac{t_{a}\rho }{2}\frac{\rho +12}{\rho^{2}+13\rho +18} & ,t_{a}\rho \ll 1,\\ \frac{8\rho +36}{\rho^{3}+15\rho^{2}+44\rho +36} & ,t_{a}\to \infty , \end{array} \end{array}$$

**Fig. 2.**
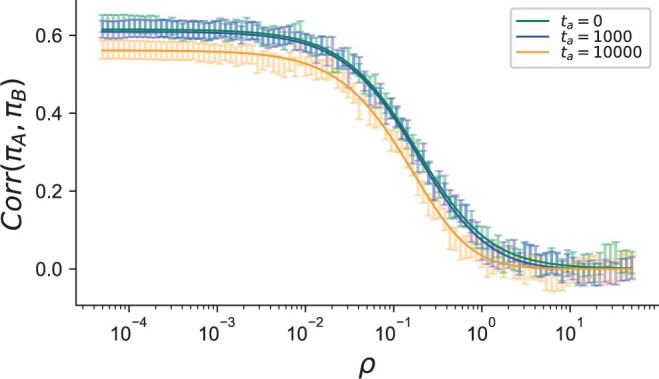
Theoretical (solid lines) and simulated correlation between pairwise differences in a constant-size demography ($N_{e}=10^{4}$) at different sample ages (in generations). Comparison of theoretical prediction of $\mathrm{Corr}(\pi_{A},\pi_{B})$ with data from 2-locus coalescent simulations with θ=0.4 (see *Methods*). Solid blue and orange lines are the theoretical predictions for $\mathrm{Corr}(\pi_{A},\pi_{B})$ from Equation (4).

As the equations show, the correlation in pairwise differences is affected by the age of the ancient sample *t_a_* in 2 ways. The first effect is due to the factor in Equation (4) that decreases as *t_a_* increases and is not dependent on *ρ*, which can be seen in Fig. 2 by the decrease for *t_a_* = 10,000 against *t_a_* = 0 for very small *ρ*. We note that the difference between *t_a_* = 10,000 and *t_a_* = 0 in Fig. 2 is more pronounced than between 1,000 and 0, because *t_a_* in Equation (4) is on the coalescent scale. The second effect occurs in how *t_a_* affects $\mathrm{Corr}(L_{A},L_{B})$ (Fig. 2). For values of $t_{a}\rho \ll 1$, the correlation decays linearly with *t_a_* and with $O(\rho^{-1})$ for *ρ*. The decay decreases more rapidly as $O(\rho^{-2})$ when $t_{a}\rho \gg 1$ and as *t_a_* gets large (the third case in Equation 5). This is because of the additional time (*t_a_*) that the recombination process has to break apart the shared genealogical history at each locus.

#### The impact of nonequilibrium demographic history on the correlation in pairwise differences

**Fig. 3.**
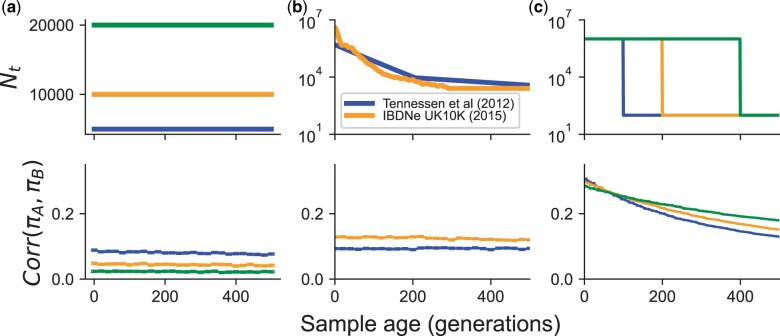
The impact of varying demographic history on the correlation in pairwise differences at 2 loci. For all simulations, the recombination rate between the loci was set to $10^{-4}$ per generation (∼10 kb, assuming 1 cM per 1 Mb). Simulated scenarios include: a) constant population size, b) inferred models of population growth, and c) models of instantaneous population growth. Each timepoint had 50,000 replicate simulations.

To explore the effects of varying population size through time, we simulated haplotype data under models of constant size, instantaneous growth, and trajectories inferred from previous studies of human populations that include both bottlenecks and growth (Tennessen *et al.* 2012; Browning and Browning 2015; Fig. 3). Motivated by how most human aDNA data are from approximately the last 15,000 years, we investigated the correlations on a timescale of 500 generations.

In models with constant population size, larger population sizes lead to smaller inter-locus correlations (lower LD). In all our simulations $\rho t_{a}\ll 1$, so on the time-scale of 500 generations, the correlation in branch length decreases linearly as expected with sampling age (Equation 4, Supplementary Fig. 2A). Across all population sizes, we observe significantly negative relationships between sample age (on the coalescent scale) and the correlation in branch length akin to what we predict in Equation (4) (for linear regression of $\mathrm{Corr}(L_{A},L_{B})∼\beta t_{a}$, we find for $N_{e}=5\times 10^{3},\hat{\beta }=-0.43;N_{e}=10^{4},\hat{\beta }=-0.52;N_{e}=2\times 10^{4},\hat{\beta }=-0.53$). The negative effect of *t_a_* on the correlation in total branch length in turn decreases the correlation in pairwise differences (Fig. 3a).

When simulating under the population size trajectories from Tennessen *et al.* (2012) or from the Browning and Browning (2015), “UK10K IBDNe model” in reference to the original dataset, the correlations are smaller than the UK10K IBDNe model, which includes a larger population size in the last few generations but an overall *N_e_* (estimated using Watterson’s estimator, see *Methods*) that is smaller than the Tennessen model ($N_{\mathrm{Tennessen}}\approx 6922.91;N_{UK10K-\mathrm{IBDNe}}\approx 2670.19$; Fig. 3b). In a linear model, the correlation in pairwise differences decreases with age under the UK10K IBDNe model [${\hat{\beta }}_{\mathrm{age}}=-0.41$, 95% CI = (−0.51, −0.31)] and not in the Tennessen *et al.* (2012) model [${\hat{\beta }}_{\mathrm{age}}=0.04$, 95% CI: (−0.03, 0.12)].

For the case of step-wise population growth (Fig. 3c), we make 3 observations. First, the decrease in the correlation in pairwise differences is no longer approximately linear with time but decays nonlinearly, with the rate of decay decreasing with sample age. Second, the correlation in pairwise differences is highest at short time-scales for the most recent growth event, and at long-timescales for the most ancient growth event. This can be interpreted again as a result of the very low *N_e_* in this setting such that the factor scaling the correlation in pairwise differences (Equation 4) dominates the behavior after $t_{a}\approx 150$ generations (when the correlation in branch length is similar across all settings). Third, the correlation in the branch length is substantially higher (>0.8) when compared with the previously inferred demographies (Supplementary Fig. 2).

The step-wise growth scenario is interesting in that due to the large, recent increase in population size, we expect roughly star-like genealogies with coalescent times concentrated around the start of the growth event (Slatkin 1996; Rosenberg and Hirsh 2003). In this scenario, we find the correlation between loci in the branch lengths is increased greatly (Supplementary Figs. 2C and 8) which contributes to elevating the $\mathrm{Corr}(\pi_{A},\pi_{B})$. At the same time, as *θ* is decreased relative to other scenarios (due to lower *N_e_*), we do not see as drastic an increase in the correlation between pairwise differences as in the branch length (Equation 4). Intuitively, as *N_e_ decreases*, the correlation in total branch length between loci *increases* as the coalescent rate increases if the recombination rate is held fixed; lowering *N_e_* also decreases *θ*, which increases the correlation in pairwise differences between loci.

Finally, we also investigated the correlation in pairwise differences in a 2-population model of divergence without gene flow. We assume the modern and ancient haplotype are each sampled from different populations. In this scenario, *both* the ancient and modern haplotypes can become uncoupled prior to any possibility of inter-haplotype coalescence lowering the expected correlation in pairwise diversity (Appendix A). In this model, we find the correlation in number of pairwise differences decreases as a function of the sum of the divergence time and the sampling time ($t_{\mathrm{div}}+t_{a}$; Supplementary Fig. 1).

### Correlation of pairwise differences in time-staggered whole-genome sequencing data

**Fig. 4.**
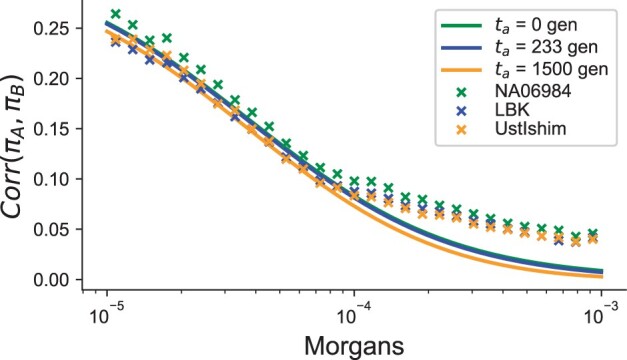
Comparison of the correlation in pairwise differences between LBK, Ust-Ishim, and a modern CEU control individual. Points represent the estimate of the pairwise correlation between randomly chosen pairs of loci (see *Methods*). When computing the theoretical curves, we used $N_{e}=10^{4}$ and a mutation rate $\mu =1.2\times 10^{-8}$ per basepair-per-generation.

Next, we explored the correlation of pairwise differences in modern and ancient human whole-genome sequencing data with 2 high-coverage samples from 2 different ages. We restricted to analyzing high-quality whole-genome sequencing data to avoid ascertainment biases and to more accurately estimate pairwise differences (see *Methods*; Fig. 4).

The first sample we chose is an ∼7,000-year-old sample from modern-day Germany associated with the Linear Ban Keramic (LBK) culture and labeled variously in previous studies as the Stuttgart *LBK* sample or simply the *LBK* sample (Lazaridis *et al.* 2014). The second sample is ∼45,000 years old and from Western Siberia, labeled *Ust-Ishim* (Fu *et al.* 2014). These samples have an order of magnitude difference in the sampling time-scale (thousands vs tens-of-thousands years).

To investigate the correspondence of our theory with empirical data, we compared the correlation in pairwise differences across our 2 empirical samples to the theoretical predictions from Equation (4). We find that for recombination rates < $10^{-4}$ Morgans, the scale and rate of decay of the empirical curves are consistent with the theoretical predictions (Fig. 4). However, there is a larger deviation between the empirical results and theoretical predictions at longer recombination distances ($>10^{-4}$), where in observed data there is an excess of correlation in pairwise differences (Fig. 4). The extended decay of $\mathrm{Corr}(\pi_{A},\pi_{B})$ that we see in real data is not present in data simulated under the model of (Tennessen *et al.* 2012; Supplementary Fig. 4A) or under a constant-sized demography (Supplementary Fig. 4B), suggesting that the extended decay is not attributable to demographic history alone and warrants further study.

### LD with time-stratified sampling

**Fig. 5.**
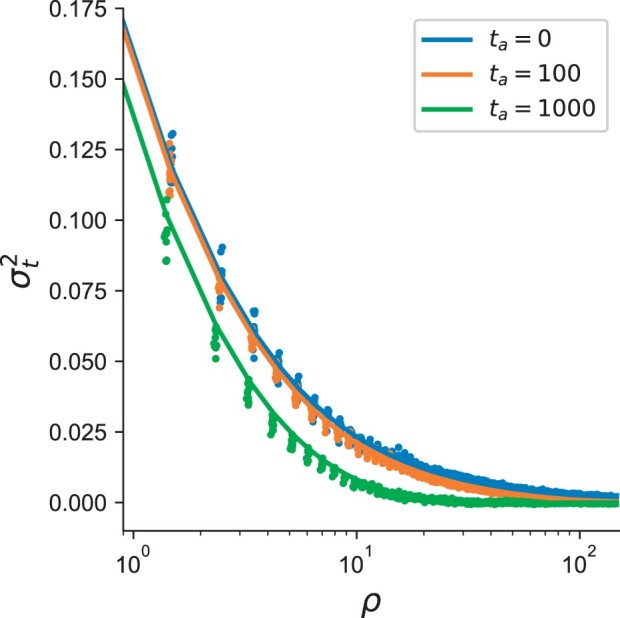
Joint product of LD between samples separated by *t_a_* generations across different population-scaled recombination rates *ρ* (see *Methods*). Dots represent results from simulation and solid lines are theoretical predictions from Equation (7).

To directly relate the joint genealogical properties described above to patterns of LD, we investigated the normalized expected product of LD (*D*) between the ancient and modern samples:

(6) $$r_{t}^{2}=\frac{D^{\left(0\right)}D^{\left(t\right)}}{p_{A}^{\left(0\right)}(1-p_{A}^{\left(t\right)})p_{B}^{\left(0\right)}(1-p_{B}^{\left(t\right)})},$$

where $p_{A}^{\left(t\right)}$ is the frequency of the derived allele at the first locus at time *t* and $D^{\left(t\right)}=p_{AB}^{\left(t\right)}-p_{A}^{\left(t\right)}p_{B}^{\left(t\right)}$ is a classic measure of LD in the sample of individuals from time *t* (Lewontin and Kojima 1960). Using the genealogical identity coefficients from McVean (2002), we derive the ratio of the expectations of the product of LD between time-points. Motivated by arguments put forth by McVean (2002) and Ragsdale and Gravel (2019) that express statistics of LD by taking the ratio of expectations (i.e. $\sigma_{d}^{2}$), we take the ratio of expectations of $r_{t}^{2}$ in Equation (6) to derive a time-stratified analog of $\sigma_{d}^{2}$. Similar to $\sigma_{d}^{2}$, we stress that our statistic $\sigma_{t}^{2}$ is not directly equivalent to $r_{t}^{2}$—is an approximation that can become poor for loci at low-frequencies McVean (2002). In Appendix B, we derive an expression for the joint product of LD across both timepoints ($\sigma_{t}^{2}$):

(7) $$\begin{array}{c} \sigma_{t}^{2}:=\frac{E[D^{\left(0\right)}D^{\left(t\right)}]}{E[p_{A}^{\left(0\right)}(1-p_{A}^{\left(t\right)})p_{B}^{\left(0\right)}(1-p_{B}^{\left(t\right)})]}\\ =\frac{\left(\rho +2\right)\left(\rho +10\right)}{\left(\rho^{3}+15\rho^{2}+48\rho +48\right)e^{\frac{t\left(\rho +2\right)}{2}}-4}, \end{array}$$

when *t *=* *0, Equation (7) reduces to the expression for $\sigma_{d}^{2}$, as shown in McVean (2002). Both simulations and our theoretical predictions show that larger time-separation between samples qualitatively decreases the joint product of LD (Fig. 5).

### The impact of time-stratified sampling in haplotype copying models

We next consider the scenario where one would be interested in modeling an ancient haplotype as a mosaic of modern haplotypes, as might arise when trying to phase or impute aDNA genotypes using a reference panel of modern haplotypes and the popular Li and Stephens haplotype copying model (Li and Stephens 2003; Song 2016). We specifically use a modified model where the recombination map positions are known *a priori* (see *Methods;*  Lawson *et al.* 2012). We focus on the maximum-likelihood estimate of the haplotype copying jump rate ($\hat{\lambda }$) for a given test haplotype as it copies off the reference panel. We view $\hat{\lambda }$ partly as a summary statistic reflecting the length scale of copying tracts and as an indicator of the expected accuracy of imputation (Stephens and Scheet 2005; Jewett *et al.* 2012).

The time-separation between the ancient haplotype and modern sample provides an opportunity for recombination events to occur among the modern reference haplotypes before the ancient lineage is able to coalesce with any individuals from the modern panel (Equation 3; Fig. 1). Thus, we expect higher jump rates as the sample age *t_a_* increases. We also expect coalescence *within* the modern panel will contribute to higher jump rates with increasing *t_a_* by effectively reducing the panel size moving farther back in time.

Using the first time coalescence between the ancient target and a member of the modern panel, we observe a saturation effect when increasing the modern panel size (Appendix C and Supplementary Fig. 5). The time until the first coalescent event involving the ancient sample is equal to the length of the external branch in the local genealogy that leads to the ancient sample, and affects the rate of recombination events that can induce switch events in the copying model. The time to the first coalescent involving the ancient sample and the modern panel decreases as a function of the reference panel size, *K*. However, as the age of the sample increases, the number of lineages extant to the reference sample becomes smaller, making the time to first coalescent event more similar across modern reference panel sizes.

**Fig. 6.**
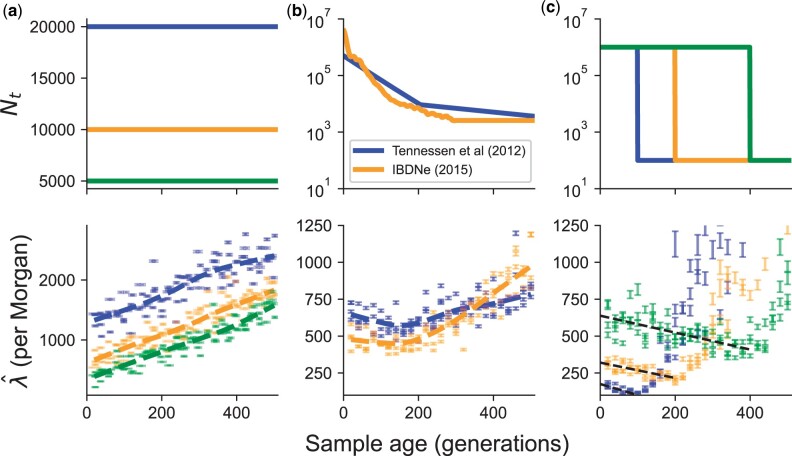
Estimation of haplotype copying jump-rate against sample age for different models of population demographic history (top row). a) constant population size, b) previously inferred models of recent population growth, and c) models of instantaneous population growth. The inferred parameters should be interpreted in terms of the average jumps per Morgan.

Using simulations with populations of constant size, we find that the realized copying jump rate indeed increases with age, and does so monotonically as a function of the age of the test haplotype under a model of constant population size (Fig. 6a). The simple monotonic relationship can break down in nonequilibrium demographic models. For instance, in demographic models including recent population growth for European populations, we find that there is an initial decrease in $\hat{\lambda }$ from the present to ∼150 generations ago before a more rapid increase moving back into the past (Fig. 6b;  Tennessen *et al.* 2012; Browning and Browning 2015). A similar result is observed more dramatically in simulations of instantaneous growth, with a common feature being a decreasing relationship between $\hat{\lambda }$ and sample age up to the time of onset of instantaneous growth, reflective of the effect of a strong conditioning on the coalescent time (Fig. 6c and  Supplementary Fig. 8).

#### Haplotype copying jump-rates in human aDNA data

To compare our simulation experiments on the dependence of the jump-rate with sampling time to empirical data, we applied our jump rate estimation to a collection of 1,159 ancient human samples (see *Methods*). To avoid potential errors introduced by statistical phasing, we analyzed only haploid carriers of the X chromosome by taking samples labeled as male in both the ancient data and the modern reference panel (1000 Genomes Project data; Auton *et al.* 2015). Thus, the analysis used 47,094 bi-allelic SNPs observed on the X chromosome. To avoid the potential effects of population structure confounding the impact of time-stratified sampling and to maximize the sample size, we focus primarily on Europe as it is the region with the highest density of aDNA samples, and we used *n *=* *49 CEU male X chromosomes to define the modern reference panel (see Supplementary Fig. 6 for experiments with alternate panels).

**Fig. 7.**
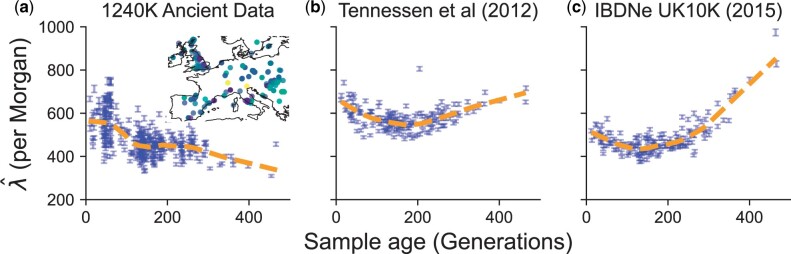
Comparison of estimated haplotype copying jump rates between real data and simulations. a) Estimate of the jump rate in ancient male X-chromosomes within 1,500 km of central Europe. b) Maximum likelihood estimates of the haplotype copying jump rate using simulated X-chromosomes under the model of Tennessen *et al.* (2012). c) Estimated jump rates using simulated data under the model of Browning and Browning (2015).

Based on copying jump rates estimated across 344 ancient male X-chromosome samples from across Europe (see *Methods* for a description of the dataset), we find that the estimated jump rate *decreases* as a function of sample age (Fig. 7a). Accounting for spatial variables (Latitude, Longitude, and Latitude × Longitude) in a linear model (see *Methods* and Supplementary Fig. 6), we find the effect of sample age on the estimated copying jump rate is negative ($\hat{\beta }=-0.54$; 95% CI = (−0.63, −0.46)]. Filtering for the highest 25% coverage individuals did not change the result (Supplementary Fig. 9). The inferred haplotype copying error rate (*ϵ*) also decreases with age, suggesting the observed decrease in *λ* is not an artifact of the inference procedure (Supplementary Fig. 10).

This decrease is contrary to our idealized simulations with constant population size (Fig. 6a) and in agreement with the simulations involving some aspect of recent growth (Fig. 6, b and c). To make the comparison more exact, we replicate simulations of Tennessen *et al.* (2012) and Browning and Browning (2015) with the exact temporal sampling structure of the real 344 samples and using a sex-averaged recombination map for the X chromosome (Kong *et al.* 2010). With these simulations, we are able to replicate an initial decrease in the jump-rate as a function of sampling time (Fig. 7, b and c). However, the simulations do not capture the duration of the decrease in jump-rate with sample age, which we find to be ≈400 generations in the real data.

## Discussion

In this article, we have developed theory to understand the effects of serial sampling on patterns of haplotype variation in the context of 2 models, the 2-locus coalescent model and the haplotype copying model. Both of these models are used to describe patterns of LD in population genetic data, and share several features with one another. Both models capture the relationship between recombination distance and the breakdown of LD, but the 2-locus genealogies consider patterns only at 2-loci whereas the haplotype copying model considers a multilocus perspective. It should also be noted that the 2-locus genealogical model explicitly considers the time of coalescent and recombination events, whereas the haplotype copying model, in the form used here, does not consider the timing of particular events. However, in spite of their differences, they both have wide relevance in that they provide theoretical results for the expected patterns of linked variation, underlying standard approaches to analyze modern haplotype data.

In the 2-locus coalecenscent, we find that with larger time-separation between samples, the correlation in branch length at 2 loci decreases by an amount proportional to the probability of uncoupling of a sampled modern haplotype over *t_a_* units of time (Equation 4). In constant-size populations and small values of $t_{a}\rho$, the decrease is linear in time. As *t_a_* increases the decay of correlation in branch lengths to occur with order $O(\rho^{-2}$) vs $O(\rho^{-1}$). Intuitively, the additional marginal branch length on which a recombination event can occur ($2+t_{a}$ vs 2 in expectation) is disrupting between-locus correlation. Demographic history also shapes the correlation in branch length between loci, with $\mathrm{Corr}(L_{A},L_{B})$ increasing as *N_e_* decreases due to a decrease in the variance in coalescent times (Supplementary Fig. 2). For larger values of *t_a_* there is an additional decrease in the correlation of pairwise differences between loci, $\mathrm{Corr}(\pi_{A},\pi_{B})$, that arises from the impact of mutations (the denominator of Equation 4). For small values of *t_a_* ($t_{a}\ll 2$ coalescent units) the correlation of branch length essentially determines the behavior of the correlation in observable number of differences between 2 loci.

The expected joint LD coefficient between data sampled at different times decreases across all recombination scales in the simulations and the theoretical derivations (Fig. 5). However, it is important to note that our simulations here represent an idealized scenario with a large number of ancient haplotypes (*n *=* *500) and no genotyping error. Therefore, it will be of further interest to determine if statistics such as the joint LD coefficient may be informative for demographic inference, while accounting for potential error modes from realistic data sources.

Our analysis of the haplotype copying rate $\hat{\lambda }$ revealed interesting impacts of demographic history. In constant-size models, the inferred copying rate increased with the sample age as one might expect due to recombination events; however, in cases of strong recent population growth (Tennessen *et al.* 2012; Reppell *et al.* 2014; Browning and Browning 2015) the inferred copying rate decreases initially with age and then increases. To understand this, consider how the haplotype copying jump-rate, $\hat{\lambda }$, is inversely related to the expected branch-length shared between an ancient haplotype and a member of the modern panel, because recombination events that occur on these branches can initiate copying-switch events (Li and Stephens 2003; Paul *et al.* 2011; Steinrücken *et al.* 2013). In cases with rapid population growth, there are initially limited numbers of coalescent events, followed by a high rate when the population is small, looking backwards in time. Samples that are sampled sequentially closer to the onset of growth have shorter branch length on which potential switch events occur, producing the initial negative relationship. For samples that are sampled *more ancestrally than* the onset of population growth, we find that the jump rate increases as the coalescent time are no longer affected by the onset of growth (Fig. 6c).

Our empirical analysis of aDNA data from western Eurasia supported a negative relationship between the haplotype copying rate and sample age. In contrast with the demographic models simulated, the empirical data show an extended decrease in the jump rate, reaching over ∼400 generations. Similar discrepancies arise when comparing the correlation in pairwise differences in empirical data (Fig. 4). We consider 2 potential explanations for the discrepancy between simulations and observations: unmodeled aspects of population demographic history not captured by existing models used for simulation or aDNA data artifacts. Throughout our experiments for both the haplotype copying rate and correlation in pairwise differences, we found that demographic models capturing more detail of recent Eurasian history did not adequately predict either statistic. However, there may still be potential unmodeled aspects of relevance to our statistics here. For example, the duration of the decrease in the estimated copying rate could be due to smaller local population sizes in the more distant past than is reflected in the models. This is particularly relevant given the time-scale of ∼400 generations (∼12,000 years) as this extends into the Mesolithic and Paleolithic eras during which populations were likely small in overall size and deeply structured (Premo and Hublin 2009; Haak *et al.* 2015; Skoglund and Mathieson 2018). If ancestral population structure existed in this period, it may have biased inferred effective population size upwards in models that were fit under the assumption of a single panmictic population (Li and Durbin 2011, Supplementary Section 1.6). We also recognize that due to population turnover, the proportion of ancestry directly ancestral to the modern reference panel may fluctuate as a function of time due to population turnover, leading to temporal patterns in the jump rate. Regarding the aDNA data, in our empirical analysis, we do not find any significant effects of coverage on the qualitative result that the jump-rate decreases as a function of time (Supplementary Fig. 9B). If error rates increase with sample age it would seem to run counter to the observed result, causing elevated jump rate estimates as one goes further back in time; however, this is not what we observe in our joint estimation (Supplementary Fig. 10). Some complex form of reference bias increasing with age and interacting with the haplotype copying model may be plausible. Overall, the result suggests there may be interesting insights to be gained by more detailed empirical analyses of haplotypic patterns in aDNA.

Many methods have been developed in the context of haplotype copying models, from imputation and phasing (e.g. Howie 2009), estimation of recombination rates (e.g. Li and Stephens 2003), to fine-scale ancestry estimation (e.g. Lawson *et al.* 2012). Our theoretical results leave important considerations for each of these application domains with serially sampled data. For imputation and phasing, the increase in the copying jump rate as a function of time under constant population sizes implies that LD will be lower in relation to the first coalescent time with a member of the modern panel, and will lower the copying accuracy at longer genetic distances (Appendix C; Jewett *et al.* 2012). For samples that are sufficiently old, there is a diminishing benefit for generating larger modern reference panels (Appendix C), which primarily results in improvements in imputation and phasing for modern samples due to recent relatedness (Jewett *et al.* 2012; McCarthy *et al.* 2016).

Our exploration of the impact of population demography (particularly population growth) and our empirical analysis of the male X chromosome paints a more optimistic picture for the analysis of human aDNA using the haplotype copying model. We find that there is a substantial attenuation of the increase in the haplotype copying jump-rate ($\hat{\lambda }$) under scenarios of recent growth, and even potential decreases in the case of instant population growth (Fig. 6). Together with our empirical result of the jump rate decreasing as a function of time across male X chromosomes in ancient European samples (Fig. 7), the results support the idea that we may be able to impute common variants relatively accurately in human populations that have undergone recent rapid growth. Indeed, the empirical accuracy of imputation is relatively high for samples within the past ∼6,000 years (Gamba *et al.* 2014; Martiniano *et al.* 2017). In addition to the “reference-based” phasing we have explored in this work, methods that iteratively sample haplotypes from the input genotypes have advantages for phasing aDNA when modern reference panels lack the haplotype and allelic diversity present in ancient samples (e.g. Rubinacci *et al.* 2020). We leave this comparison of phasing and imputation accuracy from exclusively reference-based models with the addition of iterative haplotype sampling for future work, though we expect some of the insights gained here will help this exploration.

As caveats, our theoretical results here do not account for some important features of aDNA data. Specifically, we have not attempted to model genotyping error and low-coverage data, both common in the analysis of aDNA (e.g. Dabney *et al.* 2013). Our results on pair-wise loci could be extended to directly model the effects of errors at one or both loci. Methods using haplotype copying HMMs with emission probabilities directly modeling low-coverage sequencing data (e.g. Rubinacci *et al.* 2020) are more applicable to account for this sparsity in aDNA analysis. Another caveat is that due to the wide temporal range and the absolute number of samples available (Olalde and Posth 2020), our empirical analyses focused on samples from western Eurasia. As aDNA technology improves and sampling becomes less centered on western Eurasia, it will be interesting to reanalyze the relationship between the jump-rate and sample age across multiple regions with varied demographic histories.

With the abundance of aDNA data being generated across a wide array of organisms, statistical and theoretical advances will need to similarly account for this new dimension in the data. Here, we have highlighted the impact of time-stratified sampling for 2 related models, the 2-locus coalescent with recombination and the haplotype copying model. We expect that our theoretical treatment of these models will serve to inform advances in statistical population genetic methods that account for serially sampled data to maximize their utility for inference.

## Data availability

All results in this article can be reproduced directly from repositories specified in the *Online Resources*.

Supplemental material is available at *GENETICS* online.

## Supplementary Material

iyac038_Supplementary_Data

## Figures and Tables

**Fig. A2.**
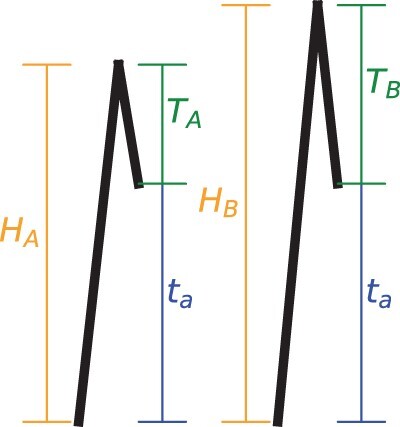
Description of variables in the 2-locus case. *H* is the total tree height, *T* is the coalescent time of the ancient and modern lineage, and *t_a_* is the sampling time of the ancient lineage (in coalescent units). Here subscripts *A*, *B* denote the 2 loci separated by scaled recombination distance *ρ*.

**Fig. A3.**
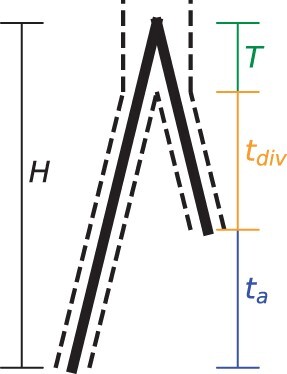
Description of variables in the single-locus case. *H* is the total tree height, *T* is the coalescent time of the ancient and modern lineage, and *t_a_* is the sampling time of the ancient lineage (in coalescent units).

**Fig. A4.**
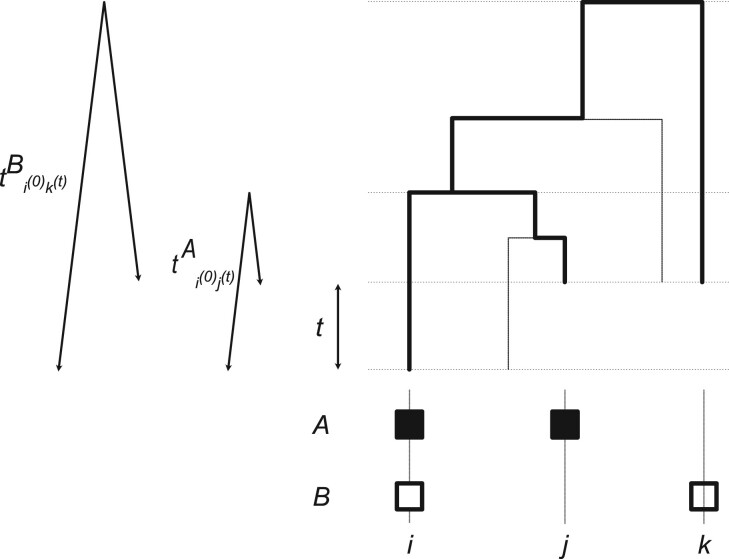
Schematic describing properties of lineages required for estimation of $E[t_{i^{\left(0\right)}j^{\left(t\right)}}^{A}t_{i^{\left(0\right)}k^{\left(t\right)}}^{B}]$ in the case with time-stratification. Figure adapted from McVean (2002) for our case of time-stratification.

## Appendix A: The 2-locus ancestral process with population continuity and ancient sampling

We first begin with a model of constant population size and where we sample 1 haplotype from the present and 1 haplotype at time *t_a_* ago (in coalescent units). The population is assumed to be constant in size with population scaled recombination rate $\rho =4N_{e}r$. Since we have 2-samples from different time-points, we have 2 phases of the process: (1) where only the modern lineage can evolve at 2 loci ($0\leq t<t_{a}$) and when both haplotypes are available to coalesce and recombine with one another ($t\geq t_{a}$). The states and possible transitions (with their corresponding rates) are shown in Fig. A1.

Before calculating *joint* moments of the genealogical properties across 2 loci, we calculate marginal moments at individual loci: (1) E[T], the time to coalesce between the 2 sequences after both are able to coalesce, (2) E[H], the height of the genealogy at a single locus, and (3) E[L], the expected total branch length at a single locus. All of these quantities are scaled by twice the population size ($2N_{e}$), which we refer to as the “coalescent scale” (see Fig. A2 for a schematic of these marginal quantities). The variable T∼Exponential(1) when both haplotypes are sampled from the same population. These marginal quantities can then be obtained in the model with time-stratified sampling as:

E[T]=Var[T]=1,

for the expectation and variance of *T*,

$$\begin{array}{c} E[H]=E[T+t_{a}]\\ =1+t_{a},\\ Var[H]=Var[T+t_{a}]=1, \end{array}$$

for the expectation and variance of *H*, and

$$\begin{array}{c} L=2H-t_{a}\\ E[L]=E[2H-t_{a}]\\ =2+t_{a},\\ Var[L]=Var[2H-t_{a}]\\ =4Var[H]=4, \end{array}$$

for the expectation and variance of *L*. Following the definition of these marginal moments, we calculate the covariance in the branch lengths at each locus, $\mathbb{C}ov(L_{A},L_{B})$, as:

$$\begin{array}{c} \mathbb{C}ov(L_{A},L_{B})=E[L_{A}L_{B}]-E[L_{A}]E[L_{B}]\\ E[L_{A}L_{B}]=E[(2H_{A}-t_{a})(2H_{B}-t_{a})]\\ =E\left[4H_{A}H_{B}-2t_{a}H_{A}-2t_{a}H_{B}+t_{a}^{2}\right]\\ =4E[H_{A}H_{B}]-4t_{a}E[H_{A}]+t_{a}^{2}\\ =4(E[T_{A}T_{B}]+2t_{a}+t_{a}^{2})-4t_{a}(1+t_{a})+t_{a}^{2}. \end{array}$$

These derivations show that we can compute $\mathbb{C}ov(L_{A},L_{B})$ under the time-staggered sampling model by computing $E[T_{A}T_{B}]$.

We approach this using a “staggered” version of the Simonsen–Churchill Model as described in the main text (Simonsen and Churchill 1997; Hobolth and Jensen 2014; Fig. A1). In the phase where $t<t_{a}$, with a single modern haplotype, we consider this as a 2-state continuous-time Markov process with the rate matrix:

$$\begin{array}{c} Q=\left[\begin{array}{cc} -\frac{\rho }{2} & \frac{\rho }{2}\\ 1 & -1 \end{array}\right], \end{array}$$

which we use to solve for the probability that the ancestral process is in state *x* at time *t_a_* as:

$$\begin{array}{c} P_{t_{a}}(x=(1,1,1))={\left(e^{Qt_{a}}\right)}_{0,1}\\ =\frac{\rho (1-e^{-t_{a}(\frac{\rho }{2}+1)})}{\rho +2}\\ P_{t_{a}}(x=(2,0,0))=1-P_{t_{a}}(x=(1,1,1)), \end{array}$$

where the state x=(2,0,0) represents 2 lineages that are ancestral to both locus *A* and locus *B* and the state x=(1,1,1) represents 1 lineage ancestral to both locus *A* and *B*, 1 lineage ancestral to locus *A*, and 1 lineage ancestral to locus *B* (Hobolth and Jensen 2014; Simonsen and Churchill 1997). This corresponds to our “uncoupled state” in the main text. The 2 states in the Markov process with a single present haplotype can only be “coupled” [(2, 0, 0)] or “uncoupled” [(1, 1, 1)].

Returning to our computation of $E[T_{A}T_{B}]$ in the second phase of the ancestral process ($t>t_{a}$), we obtain:

(8) $$\begin{array}{c} E_{\left(2,0,0\right)}[T_{A}T_{B}]=\frac{\rho^{2}+14\rho +36}{\rho^{2}+13\rho +18},\\ E_{\left(1,1,1\right)}[T_{A}T_{B}]=\frac{\rho^{2}+13\rho +24}{\rho^{2}+13\rho +18},\\ E[T_{A}T_{B}]=P_{t_{a}}(x=(2,0,0))E_{\left(2,0,0\right)}[T_{A}T_{B}]\\ \;\;+P_{t_{a}}(x=(1,1,1))E_{\left(1,1,1\right)}[T_{A}T_{B}]\\ =\left(1-\frac{\rho (1-e^{-t(\frac{\rho }{2}+1)})}{\rho +2}\right)\frac{\rho^{2}+14\rho +36}{\rho^{2}+13\rho +18}\\ \;\;+\frac{\rho (1-e^{-t(\frac{\rho }{2}+1)})}{\rho +2}\frac{\rho^{2}+13\rho +24}{\rho^{2}+13\rho +18}, \end{array}$$

where $E_{x}$ indicates the expectation conditional on starting in state *x* of the ancestral process. The first 2 expressions above are derived in Durrett (2008, Chapter 3), where both haplotypes are sampled at present. The last expression is a weighting of the expectations from different starting states in the 2-locus ancestral process, where the weight corresponds to the probabilities that the modern haplotype is uncoupled at the time the ancient haplotype is sampled, *t_a_*. From this we can compute the covariance in the branch length, $\mathbb{C}ov(L_{A},L_{B})$ and $\mathbb{C}\mathrm{orr}(L_{A},L_{B})$: by substituting the Equation (8) into the relevant expressions previously defined, leading to the expression:

(9) $$\begin{array}{c} \mathbb{C}\mathrm{orr}(L_{A},L_{B})=\frac{\mathbb{C}ov(L_{A},L_{B})}{\sqrt{\mathrm{Var}(L_{A})\mathrm{Var}(L_{B})}}\\ =E[T_{A}T_{B}]-1, \end{array}$$

which simplifies to Equation (4) in the main text. The lower and upper limits of *t_a_* are 0 and ∞, and we show the asymptotic behavior of $\mathrm{Corr}(L_{A},L_{B})$ in terms of *ρ*:

$$\begin{array}{c} \frac{2}{\rho +2}<P(x=(2,0,0))\leq 1,\forall t_{a}\in [0,\infty )\\ \mathrm{Corr}(L_{A},L_{B})=E[T_{A}T_{B}]-1\\ \mathrm{Corr}(L_{A},L_{B})|t_{a}\to 0=\frac{\rho^{2}+14\rho +36}{\rho^{2}+13\rho +18}-1\\ =\frac{\rho +18}{\rho^{2}+13\rho +18}\\ \mathrm{Corr}(L_{A},L_{B})|t_{a}\to \infty =\frac{2}{\rho +2}\frac{\rho^{2}+14\rho +36}{\rho^{2}+13\rho +18}\\ \;\;+\frac{\rho }{\rho +2}\frac{\rho^{2}+13\rho +24}{\rho^{2}+13\rho +18}-1\\ =\frac{8\rho +36}{\rho^{3}+15\rho^{2}+44\rho +36}. \end{array}$$

This derivation highlights the change in the rate of decay in the correlation of the branch length as a function of the sampling time from $O(\rho^{-1})$ to $O(\rho^{-2})$.

To relate the correlation in total branch length to the correlation in the number of pairwise differences between 2 sequences, we use the following identities for the case where mutations occur as a Poisson process with rate θ/2 along branches, where *θ* is the population-scaled mutation rate $\left(\theta =4N_{e}\mu \right)$ (Hobolth *et* al. 2019):

$$\begin{array}{c} \pi_{A}|L_{A}∼\text{Pois}\left(\frac{\theta }{2}L_{A}\right),\\ \pi_{B}|L_{B}∼\text{Pois}\left(\frac{\theta }{2}L_{B}\right),\\ E[\pi_{A}]=E[\pi_{B}]=E[E[\pi_{A}|L_{A}]]=\frac{\theta }{2}E[L_{A}],\\ \mathrm{Var}(\pi_{A})=E[\mathrm{Var}(\pi_{A}|L_{A})]+\mathrm{Var}(E[\pi_{A}|L_{A}]),\\ =\frac{\theta }{2}E[L_{A}]+{\left(\frac{\theta }{2}\right)}^{2}Var(L_{A})\\ E[\pi_{A}\pi_{B}]=E[E[\pi_{A}\pi_{B}|L_{A}L_{B}]]=\frac{\theta^{2}}{4}E[L_{A}L_{B}],\\ \mathbb{C}ov(\pi_{A},\pi_{B})=E[\pi_{A}\pi_{B}]-E[\pi_{A}]E[\pi_{B}]=\frac{\theta^{2}}{4}Cov(L_{A},L_{B}),\\ \mathbb{C}\mathrm{orr}(\pi_{A},\pi_{B})=\frac{\mathrm{Cov}(\pi_{A},\pi_{B})}{\sqrt{\mathrm{Var}(\pi_{A})\mathrm{Var}(\pi_{B})}},\\ =\frac{\frac{\theta^{2}}{4}Cov(L_{A},L_{B})}{\sqrt{{\left(\frac{\theta }{2}E[L_{A}]+\frac{\theta^{2}}{4}Var(L_{A})\right)}^{2}}}\\ =\frac{\mathbb{C}ov(L_{A},L_{B})}{\frac{2}{\theta }E[L_{A}]+\mathrm{Var}(L_{A})}\\ =\frac{1}{1+\frac{2+t_{a}}{2\theta }}(E[T_{A}T_{B}]-1), \end{array}$$

leading to a relationship with the correlation in the branch length at each locus, $\mathbb{C}\mathrm{orr}(L_{A},L_{B})$:

(10) $$\mathbb{C}\mathrm{orr}(\pi_{A},\pi_{B})=\frac{1}{1+\frac{2+t_{a}}{2\theta }}\mathbb{C}\mathrm{orr}(L_{A},L_{B}),$$

which is Equation (4) in the main text.

### The 2-locus ancestral process with population divergence and time-stratified sampling

In this section, we assume a model with divergence between the populations containing the ancient lineage and the modern lineage at the coalescent scaled time, *t_div_*. We can partition the ancestral process into 3 phases: (1) when the modern lineage is the only 1 evolving, (2) when the ancient lineage and the modern lineage are both evolving *but are not able* to coalescent with one another, and (3) when both lineages are in the ancestral population and can coalesce with each other. These 3 phases can be seen Fig. A3.

The model with population divergence has an additional parameter, *t_div_*, the divergence time of the 2 populations. We first show the properties of the marginal tree under the divergence model (see Fig. A3, for a definition of the quantities):

$$\begin{array}{c} E[T]=\mathrm{Var}[T]=1,\\ E[H]=E[T+t_{a}+t_{\mathrm{div}}]\\ =1+t_{a}+t_{\mathrm{div}},\\ \mathrm{Var}[H]=\mathrm{Var}[T+t_{a}+t_{\mathrm{div}}]\\ =1,\\ E[L]=E[2H-t_{a}]\\ =2E[H]-t_{a}\\ =2(1+t_{a}+t_{\mathrm{div}})-t_{a}\\ =2+t_{a}+2t_{\mathrm{div}},\\ \mathrm{Var}[L]=\mathrm{Var}[2H-t_{a}]\\ =4Var[H]=4, \end{array}$$

where *t_div_* is the population divergence times in coalescent units, *t_a_* is the sampling time of the ancient lineage, *T* is the exponentially distributed time after both lineages are able to coalesce that they coalesce with one another. Using these results, we can calculate moments of the joint distribution of genealogical properties like the tree height (*H*), and total branch length (*L*). Specifically, the 2-locus ancestral process behaves independently within each population for time *t_a_* and *t_div_* and each population is assumed to have the same population size. We begin by deriving the joint expectation of tree-height $H_{A}H_{B}$:

$$\begin{array}{c} E[H_{A}H_{B}]=E[(T_{A}+t_{a}+t_{\mathrm{div}})(T_{B}+t_{a}+t_{\mathrm{div}})]\\ =E[T_{A}T_{B}]+2t_{\mathrm{div}}+2t_{a}+{\left(t_{a}+t_{\mathrm{div}}\right)}^{2}, \end{array}$$

and joint tree length $L_{A}L_{B}$:

$$\begin{array}{c} E[L_{A}L_{B}]=E[(2H_{A}-t_{a})(2H_{B}-t_{a})]\\ =4E[H_{A}H_{B}]-4t_{a}+t_{a}^{2}, \end{array}$$

where we must solve for the joint expectation of $E[T_{A}T_{B}]$, but with the additional complication of population divergence. In order to do this we must calculate the probability of being in 1 of 3 starting states at time $t_{a}+t_{\mathrm{div}}$: (1) the state x=(2,0,0) where both the ancient and modern haplotypes are “coupled,” (2) the state x=(0,2,2) where both the ancient and modern haplotype are “uncoupled,” which is possible due to the independent evolution of both lineages during $t_{a}<t<t_{a}+t_{\mathrm{div}}$, and (3) state x=(1,1,1) where one haplotype is uncoupled, whereas the other is coupled. We consider the 2 independent processes within each population until the divergence time and calculate the probabilities of being in each starting state as follows:

$$\begin{array}{c} P(x=(2,0,0)|t_{a},t_{\mathrm{div}})=P(x_{1}=(1,0,0)|t_{a}+t_{\mathrm{div}})P(x_{2}=(1,0,0)|t_{\mathrm{div}})\\ =\frac{\rho e^{-(t_{a}+t_{\mathrm{div}})(\rho /2+1)}+2}{\rho +2}\frac{\rho e^{-t_{\mathrm{div}}(\rho /2+1)}+2}{\rho +2},\\ P(x=(0,2,2)|t_{a},t_{\mathrm{div}})=P(x_{1}=(0,1,1)|t_{a}+t_{\mathrm{div}})P(x_{2}=(0,1,1)|t_{\mathrm{div}})\\ =\frac{\rho (1-e^{-(t_{a}+t_{\mathrm{div}})(\rho /2+1)})}{\rho +2}\frac{\rho (1-e^{-t_{\mathrm{div}}(\rho /2+1)})}{\rho +2}, \end{array}$$

and

$$\begin{array}{c} P(x=(1,1,1)|t_{a},t_{\mathrm{div}})=P(x_{1}=(1,0,0)|t_{a}+t_{\mathrm{div}})P(x_{2}=(0,1,1)|t_{\mathrm{div}})\\ \;\;+P(x_{1}=(0,1,1)|t_{a}+t_{\mathrm{div}})P(x_{2}=(1,0,0)|t_{\mathrm{div}})\\ =\left(\frac{\rho e^{-(t_{a}+t_{\mathrm{div}})(\rho /2+1)}+2}{\rho +2}\frac{\rho (1-e^{-t_{\mathrm{div}}(\rho /2+1)})}{\rho +2}\right)\\ \;\;+\left(\frac{\rho (1-e^{-(t_{a}+t_{\mathrm{div}})(\rho /2+1)})}{\rho +2}\frac{\rho e^{-t_{\mathrm{div}}(\rho /2+1)}+2}{\rho +2}\right). \end{array}$$

From these probabilities, we calculate the expectation of the joint coalescent times conditional on being in a specified state at time $t_{a}+t_{\mathrm{div}}$ is obtained as:

$$\begin{array}{c} E[T_{A}T_{B}]=\sum_{x\in \{(1,1,1),(2,0,0),(0,2,2)\}}P(x=x|t_{a},t_{\mathrm{div}})E_{x}[T_{A}T_{B}], \end{array}$$

where each of $E_{x}[T_{A}T_{B}]$ is defined using previously derived results under the 2-locus ancestral process conditional on being in a starting state *x* (Simonsen and Churchill 1997; Durrett 2008; Chapter 3). This is different from the model under population continuity (where the x=(0,2,2) state was not possible). If we set *t_div_* = 0, then this corresponds exactly to the model without population divergence. While the underlying mathematical results are more involved, they provide insights on how population divergence affects joint coalescent times.

We can now compute joint statistics (e.g. correlation) of the tree properties at each of the loci following common formulas, for example for the correlation in total branch length at each locus:

$$\mathbb{C}\mathrm{orr}(L_{A},L_{B})=E[T_{A}T_{B}]-1.$$

### Expectations of joint coalescent times under the time-stratified model

We assume that the following results on the joint coalescent times for 2 contemporary haplotypes starting in the same state in the 2-locus ancestral process as defined in Durrett (2008, Chapter 3) are known:

$$\begin{array}{c} E_{0}[T_{A}T_{B}|x=(2,0,0)]=\frac{\rho^{2}+14\rho +36}{\rho^{2}+13\rho +18}\\ E_{0}[T_{A}T_{B}|x=(1,1,1)]=\frac{\rho^{2}+13\rho +24}{\rho^{2}+13\rho +18}\\ E_{0}[T_{A}T_{B}|x=(0,2,2)]=\frac{\rho^{2}+13\rho +22}{\rho^{2}+13\rho +18}, \end{array}$$

and now, we will go through the individual cases for the time-stratified case: (1) both modern and ancient haplotypes start coupled, (2) both modern and ancient haplotypes are “uncoupled,” and finally (3) where *only one* of the modern and ancient haplotypes are coupled (the other is uncoupled).

We first define 2 quantities, called *γ* and *η*. The variable *γ* refers to the probability of starting in the coupled [(1, 0, 0)] state and ending in the uncoupled state [(0, 1, 1)] at time *t_a_* for a single haplotype (which is Equation 3 in the main text). The variable *η* is the converse, the probability of starting in the uncoupled state and ending in the coupled state at time *t_a_*. Using the matrix exponential $e^{-Qt_{a}}$ of the following rate matrix for the process with a single haplotype:

$$\begin{array}{c} Q=\left[\begin{array}{cc} -\frac{\rho }{2} & \frac{\rho }{2}\\ 1 & -1 \end{array}\right], \end{array}$$

we arrive at the following expressions for *γ* and *η*:

$$\begin{array}{c} \gamma =\frac{\rho (1-e^{-t_{a}(\frac{\rho }{2}+1)})}{\rho +2},\\ \eta =\frac{2(1-e^{-t_{a}(\frac{\rho }{2}+1)})}{\rho +2}. \end{array}$$

With these in hand we can start tackling our first case (1) from above:

(11) $$\begin{array}{c} E_{t_{a}}[T_{A}T_{B}|x_{t_{a}}=(1,0,0),x_{0}=(1,0,0)]=(1-\gamma )E_{0}[T_{A}T_{B}|x=(2,0,0)]\\ \;\;+\gamma E_{0}[T_{A}T_{B}|x=(1,1,1)], \end{array}$$

where, $x_{0}=(1,0,0)$ indicates that the modern haplotype is coupled, and $x_{t_{a}}=(1,0,0)$ indicates that the ancient haplotype is coupled as well. This holds because the modern haplotype can be coupled with probability 1−γ leading to state x=(2,0,0) for the joint ancestral process, or it can be uncoupled with probability *γ* resulting in state x=(1,1,1). For case (2) (both haplotypes uncoupled), we obtain:

(12) $$\begin{array}{c} E_{t_{a}}[T_{A}T_{B}|x_{t_{a}}=(0,1,1),x_{0}=(0,1,1)]=(1-\eta )E_{0}[T_{A}T_{B}|x=(0,2,2)]\\ \;\;+\eta E_{0}[T_{A}T_{B}|x=(1,1,1)]. \end{array}$$

The final case (3) is the most complicated and we break this into a further 2 subcases below:

(13) $$\begin{array}{c} E_{t_{a}}[T_{A}T_{B}|x_{t_{a}}=(1,0,0),x_{0}=(0,1,1)]=(1-\eta )E_{0}[T_{A}T_{B}|x=(1,1,1)]\\ \;\;+\eta E_{0}[T_{A}T_{B}|x=(2,0,0)],\\ E_{t_{a}}[T_{A}T_{B}|x_{t_{a}}=(0,1,1),x_{0}=(1,0,0)]=(1-\gamma )E_{0}[T_{A}T_{B}|x=(1,1,1)]\\ \;\;+\gamma E_{0}[T_{A}T_{B}|x=(0,2,2)], \end{array}$$

where the first case corresponds to the modern haplotype starting in the “uncoupled” state (denoted by the *x*_0_ in the expectation) and the second case corresponds to the modern haplotype starting in the “coupled” state.

## Appendix B: The expected product of LD between time-stratified samples

Here, we derive the scaled product of LD between time-stratified samples normalized by the heterozygosity across both sites and time points. We first start from the definition of the statistic in terms of haplotype and allele frequencies in the ancient and modern samples:

(14) $$\begin{array}{c} \sigma_{d}^{2}=\frac{E[D^{\left(0\right)}D^{\left(t\right)}]}{E\left[p_{A}^{0}(1-p_{A}^{\left(t\right)})p_{B}^{\left(0\right)}(1-p_{B}^{\left(t\right)})\right]}\\ =\frac{E\left[\left(p_{AB}^{\left(0\right)}-p_{A}^{\left(0\right)}p_{B}^{\left(0\right)})(p_{AB}^{\left(t\right)}-p_{A}^{\left(t\right)}p_{B}^{\left(t\right)}\right)\right]}{E\left[p_{A}^{\left(0\right)}(1-p_{A}^{\left(t\right)})p_{B}^{\left(0\right)}(1-p_{B}^{\left(t\right)})\right]}, \end{array}$$

where $p_{AB}^{\left(t\right)}$ is the frequency of the haplotype with the derived alleles at both loci at time *t*, $p_{A}^{\left(t\right)}$ is the frequency of the derived allele at the first locus, and $p_{B}^{\left(t\right)}$ is the frequency of the derived allele at the second locus. Using the approach of McVean (2002) we define this ratio using branch lengths in the genealogy relating modern and ancient samples, where a mutation would result in a observed pattern of identity by state (Fig. A4). We first expand the numerator as follows:

$$\begin{array}{c} E[D^{\left(0\right)}D^{\left(t\right)}]=E[(p_{AB}^{0}-p_{A}^{0}p_{B}^{0})(p_{AB}^{t}-p_{A}^{t}p_{B}^{t})]\\ =E[p_{AB}^{\left(0\right)}p_{AB}^{\left(t\right)}]-E[p_{AB}^{\left(0\right)}p_{A}^{\left(t\right)}p_{B}^{\left(t\right)}]-E[p_{A}^{\left(0\right)}p_{B}^{\left(0\right)}p_{AB}^{\left(t\right)}]+E[p_{A}^{\left(0\right)}p_{B}^{\left(0\right)}p_{A}^{\left(t\right)}p_{B}^{\left(t\right)}],\\ \approx \frac{E[I_{i^{\left(0\right)}j^{\left(t\right)}}^{A}I_{i^{\left(0\right)}j^{\left(t\right)}}^{B}]-E[I_{i^{\left(0\right)}j^{\left(t\right)}}^{A}I_{i^{\left(0\right)}k^{\left(t\right)}}^{B}]-E[I_{i^{\left(0\right)}j^{\left(t\right)}}^{A}I_{k^{\left(0\right)}j^{\left(t\right)}}^{B}]+E[I_{i^{\left(0\right)}j^{\left(t\right)}}^{A}I_{k^{\left(0\right)}l^{\left(t\right)}}^{B}]}{E[L^{A}L^{B}]}, \end{array}$$

where i,j,k,l denote sampled haplotypes. Furthermore,$I_{\left(i^{\left(0\right)}j^{\left(t\right)}\right)}^{x}$ is the branch length leading from the *T_mrca_* of the samples $i^{\left(0\right)}$ at time 0 and $j^{\left(t\right)}$ at time *t* to the *T_mrca_* of the total population (including the ancient individuals) at locus *x*. $E[L^{A}L^{B}]$ is the joint expectation of the total genealogical branch length for the complete population at both loci. The approximation in the final step above follows from assuming a small mutation rate (McVean 2002). We use the definition $I_{i^{\left(0\right)}j^{\left(t\right)}}^{A}=T^{A}-t_{i^{\left(0\right)}j^{\left(t\right)}}^{A}$, where *T^A^* is the *T_mrca_* for the total population (modern and ancient) at locus A and $t_{i^{\left(0\right)}j^{\left(t\right)}}^{A}$ is the pairwise coalescent time for samples $i^{\left(0\right)},j^{\left(t\right)}$ at locus A. Using this relationship between coalescent times and identity coefficients, we arrive at:

$$\begin{array}{c} E[D^{\left(0\right)}D^{\left(t\right)}]=E[(T^{A}-t_{i^{\left(0\right)}j^{\left(t\right)}}^{A})(T^{B}-t_{i^{\left(0\right)}j^{\left(t\right)}}^{B})]-E[(T^{A}-t_{i^{\left(0\right)}j^{\left(t\right)}}^{A})(T^{B}-t_{i^{\left(0\right)}k^{\left(t\right)}}^{B})]\\ -E[(T^{A}-t_{i^{\left(0\right)}j^{\left(t\right)})}^{A})(T^{B}-t_{\left(k^{\left(0\right)}j^{\left(t\right)}\right)}^{B})]+E[(T^{A}-t_{i^{\left(0\right)}j^{\left(t\right)}}^{A})(T^{B}-t_{k^{\left(0\right)}l^{\left(t\right)}}^{B})]\\ =\frac{E[t_{i^{\left(0\right)}j^{\left(t\right)}}^{A}t_{i^{\left(0\right)}j^{\left(t\right)}}^{B}]-E[t_{i^{\left(0\right)}j^{\left(t\right)}}^{A}t_{i^{\left(0\right)}k^{\left(t\right)}}^{B}]-E[t_{i^{\left(0\right)}k^{\left(t\right)}}^{A}t_{i^{\left(0\right)}j^{\left(t\right)}}^{B}]+E[t_{i^{\left(0\right)}j^{\left(t\right)}}^{A}t_{k^{\left(0\right)}l^{\left(t\right)}}^{B}]}{E[L^{A}L^{B}]}, \end{array}$$

where the product of pairwise coalescent times at one locus and the total *T_mrca_* at the other locus (e.g. $E[T^{1}t_{\left(i^{\left(0\right)}j^{\left(0\right)}\right)}^{2}]$) do not depend on the indices *i*, *j* (Durrett 2008; Chapter 3). This means that the numerator of the expression above can be computed using the expectations of pairwise coalescent times in the time-stratified model.

The denominator of our expression ($E[p_{A}^{\left(0\right)}(1-p_{A}^{\left(t\right)})p_{B}^{\left(0\right)}(1-p_{B}^{\left(t\right)})]$) is the probability of drawing 2 haplotypes at the first locus that are at different time points and differ in their allelic identity, *and* drawing 2 haplotypes at the second locus from different timepoints that also differ in their allelic identity. This is a measure of the time-stratified joint heterozygosity at both sites. We note that this is different from the interpretation of E[p(1−p)q(1−q)] which is the probability of a difference at the first locus and a difference at the second locus under a random draw from of a sample from a contemporary population and is the denominator of $\sigma_{d}^{2}$ (McVean 2002). We define the denominator similarly using pairwise coalescent times as:

$$\begin{array}{c} E[p_{A}^{\left(0\right)}(1-p_{A}^{\left(t\right)})p_{B}^{\left(0\right)}(1-p_{B}^{\left(t\right)})]\approx \frac{E[t_{i^{\left(0\right)}j^{\left(t\right)}}^{A}t_{k^{\left(0\right)}l^{\left(t\right)})}^{B}]}{E[L^{A}L^{B}]}, \end{array}$$

where we see that joint total branch length term $E[L^{A}L^{B}]$ will cancel out when evaluating the ratio. We can now turn to actually computing this expression using the joint expectations for coalescent times calculated in our time-stratified model (see Appendix A for the derivation of these joint coalescent times):

$$\begin{array}{c} \frac{E[D^{\left(0\right)}D^{\left(t\right)}]}{E[p_{A}^{\left(0\right)}(1-p_{A}^{\left(t\right)})p_{B}^{\left(0\right)}(1-p_{B}^{\left(t\right)})]}\\ =\frac{1}{E[T_{A}T_{B}|x_{t_{a}}=(0,1,1),x_{0}=(0,1,1)]}[E[T_{A}T_{B}|x_{t_{a}}=(1,0,0),x_{0}=(1,0,0)]\\ -E[T_{A}T_{B}|x_{t_{a}}=(0,1,1),x_{0}=(1,0,0)]\\ -E[T_{A}T_{B}|x_{t_{a}}=(1,0,0),x_{0}=(0,1,1)]\\ +E[T_{A}T_{B}|x_{t_{a}}=(0,1,1),x_{0}=(0,1,1)]], \end{array}$$

which can be simplified to the following expression after substituting the proper expressions for the joint coalescent times derived in Appendix A:

$$\frac{\left(\rho +2\right)\left(\rho +10\right)}{\rho^{3}e^{\frac{t\left(\rho +2\right)}{2}}+15\rho^{2}e^{\frac{t\left(\rho +2\right)}{2}}+48\rho e^{\frac{t\left(\rho +2\right)}{2}}+48e^{\frac{t\left(\rho +2\right)}{2}}-4},$$

which is the expression reported in the main text (Equation 7). Importantly, we find that when *t *=* *0, the expression simplifies to $\frac{\rho +10}{\rho^{2}+13\rho +22}$ which is the expression for $\sigma_{d}^{2}$ in the case with 2 contemporary samples (McVean 2002).

## Appendix C: Expected-time to first coalescent for an ancient sample

Here, we consider a single ancient haplotype sampled at a time *t_a_* in the past and how it coalesces into the ancestral lineages of a reference panel of size *K* haplotypes sampled at the present. We define the random variable $T^{*}$ as the additional time of a coalescent event involving the ancient haplotype and a lineage ancestral to the modern reference panel after the time that the ancient haplotype is sampled (*t_a_*). The expectation of this quantity can be written as:

$$\begin{array}{c} E_{t_{a},K}[T^{*}]=E[E[T^{*}|A_{K}(t_{a})]]\\ =E\left[E\left[\sum_{j=2}^{A_{K}(t_{a})+1}P(I_{j})\sum_{i=A_{K}(t_{a})+1}^{j}T_{i}\right]|A_{K}(t_{a})\right], \end{array}$$

where $A_{K}(t_{a})$ is the number of lineages ancestral to the modern reference panel at time *t_a_*, $P(I_{j})$ is the probability that the *j*th coalescent event involves the ancient lineage, and *T_i_* is the *i*th intercoalescent time.

Starting at time *t_a_* with *n_t_* lineages, we calculate the probability that the *j*th coalescent event involves the ancient lineage as:

$$\begin{array}{c} P(I_{j})=\left(1-\frac{\left(\begin{array}{c} j-1\\ 2 \end{array}\right)}{\left(\begin{array}{c} j\\ 2 \end{array}\right)}\right)\prod_{k=A_{n}(t_{a})}^{j+1}\frac{\left(\begin{array}{c} k-1\\ 2 \end{array}\right)}{\left(\begin{array}{c} k\\ 2 \end{array}\right)}\\ =\frac{2}{j}\prod_{k=A_{n}(t_{a})}^{j+1}\left(1-\frac{2}{k}\right). \end{array}$$

In a constant population size model, we have $E[T_{j}]=\frac{2}{j(j-1)}$. Using this fact, the expected time until the first coalescence involving the ancient lineage ($T^{*}$) is:

$$\begin{array}{c} E[T^{*}|A_{K}(t_{a})]=E\left[\sum_{j=2}^{A_{K}(t_{a})+1}P(I_{j})\sum_{i=A_{K}(t_{a})+1}^{j}T_{i}\right]\\ =\sum_{j=A_{K}(t_{a})+1}^{2}\left[\frac{2}{j}\prod_{k=A_{K}(t_{a})+1}^{j+1}\left(1-\frac{2}{k}\right)\sum_{i=A_{K}(t_{a})+1}^{j}\frac{2}{i(i-1)}\right], \end{array}$$

and considering the summation over $A_{K}(t_{a})$, we arrive at our final expression:

(15) $$\begin{array}{c} E_{t_{a},K}[T^{*}]=E[E[T^{*}|A_{K}(t_{a})]]\\ =\sum_{a=K}^{1}P(A_{K}(t_{a})=a)\left[\sum_{j=a+1}^{2}\left[\frac{2}{j}\prod_{k=a+1}^{j+1}\left(1-\frac{2}{k}\right)\sum_{i=a+1}^{j}\frac{2}{i(i-1)}\right]\right]. \end{array}$$

The probability distribution $P(A_{K}(t)=a)$ involves a number of alternating sums and leads rapidly to numerical error as the sample size gets large (see Equation 15 in Chen and Chen 2013). To alleviate this issue, following Jewett and Rosenberg (2014) we approximate $P(A_{K}(t)=a)$ as $\delta (A_{K}(t)=E[A_{K}(t)])$. That is, rather than calculate the probability distribution of $A_{K}(t)$ across states 1…K, we will approximate it with its expectation $E[A_{K}(t)]$. One approximation for $E[A_{K}(t)]$ is found in Griffiths (1984):

$$E[A_{K}(t)]\approx \frac{K}{K+(1-K)e^{-t}}.$$

Further approximations for this expectation exist and are explored in greater detail in Jewett and Rosenberg (2014). We chose the above approximation largely for computational convenience as it does not involve any summation, has a simple form, and is comparably accurate when compared with other approximations (Jewett and Rosenberg 2014).

The additional time to coalescence for the ancient sample ($E[T^{*}]$) is proportional to the number of recombination events that can affect the genealogical closest haplotype to the ancient sample that is in the modern panel. For example, for a sample with $t_{a}=2\times 10^{-4}$ there is $E[T^{*}]\approx 2\times 10^{-3}$ and $2\times 10^{-4}$ with a panel size of *K *=* *1,000 and 10,000, respectively (Supplementary Fig. 5). This guides the intuition that for large panel sizes and recent sampling times, the time for the ancient haplotype to coalesce with the panel is quite small, and therefore we expect the haplotype copying rate to be fairly small (leading to longer shared blocks). This is the key intuition behind long-range phasing methods that take advantage of recent relatedness (e.g. Loh *et al.* 2016). For samples on the order of $∼10^{-2}$ coalescent units, the relative ratio is 1.17 for $E[T^{*}]$ with modern panel sizes of *K *=* *1,000 and *K *=* *10,000 (as opposed to 6.99 when $t_{a}=10^{-4}$). This highlights a saturation effect of within-panel coalescence at deeper times, limiting the expected utility of large modern panels for the setting with substantially ancient samples (Supplementary Fig. 5).
