# Supplementary material for: Properties of 2-locus genealogies and linkage disequilibrium in temporally structured samples
Source: Genetics. 2022 Mar 16;221(1):iyac038. doi: 10.1093/genetics/iyac038 (PMC9245597; doi:10.1093/genetics/iyac038)
Supplement: iyac038_Supplementary_Data [file iyac038_supplementary_data.pdf]

Appendices

Appendix 1: The Two-Locus Ancestral Process with Population Continuity and Ancient Sampling

We first begin with a model of constant population size and where we sample one haplotype from the present and one haplotype at time t_a ago (in coalescent units). The population is assumed to be constant in size with population scaled recombination rate $\rho = 4N_e r$. Since we have two-samples from different time-points, we have two phases of the process: (1) where only the modern lineage can evolve at two loci ($0 \leq t < t_a$) and when both haplotypes are available to coalesce and recombine with one another ($t \geq t_a$). The states and possible transitions (with their corresponding rates) are shown in Figure A1.

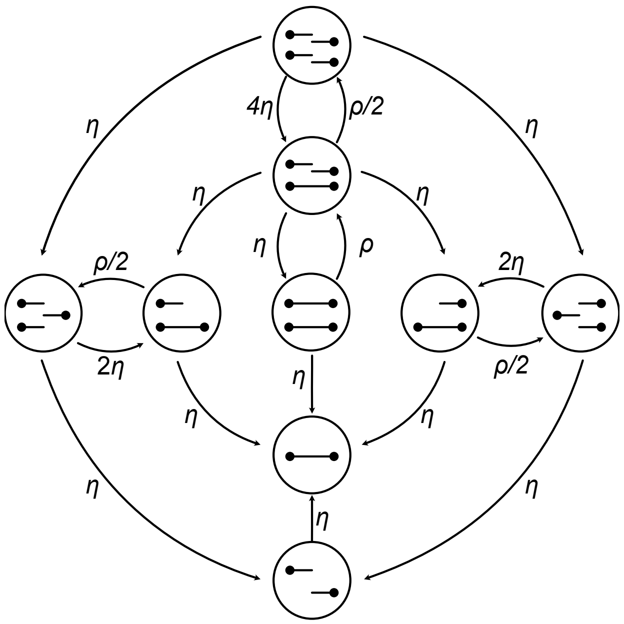

Figure A1 Markov chain model for the ancestral process at two loci from Simonsen and Churchill (1997). In all settings for two modern haplotypes we assume that we start from the state in the middle (state "0") in all applications, which means that all sampled haplotypes are coupled. The parameter η represents the coalescent rate and the parameter ρ represents the recombination rate (measured in coalescent units). Figure adapted from Hobolth and Jensen (2014).

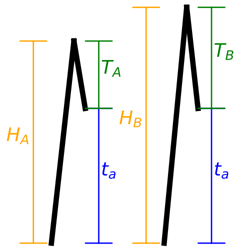

Figure A2 Description of variables in the two-locus case. H is the total tree height, T is the coalescent time of the ancient and modern lineage, and t_a is the sampling time of the ancient lineage (in coalescent units). Here subscripts A, B denote the two loci separated by scaled recombination distance ρ.

Before calculating *joint* moments of the genealogical properties across two loci, we calculate marginal moments at individual loci: (1) $E[T]$, the time to coalesce between the two sequences after both are able to coalesce, (2) $\mathbb{E}[H]$, the height of the genealogy at a single locus, and (3) $\mathbb{E}[L]$, the expected total branch length at a single locus. All of these quantities are scaled by twice the population size ($2N_e$), which we refer to as the "coalescent scale" (see Figure A2 for a schematic of these marginal quantities). The variable $T \sim \text{Exponential}(1)$ when both haplotypes are sampled from the same population. These marginal quantities can then be obtained in the model with time-stratified sampling as:

$$\mathbb{E}[T] = \mathbb{V}ar[T] = 1,$$

for the expectation and variance of T,

$$\begin{aligned}\mathbb{E}[H] &= \mathbb{E}[T + t_a] \\ &= 1 + t_a, \\ \mathbb{V}ar[H] &= \mathbb{V}ar[T + t_a] = 1,\end{aligned}$$

for the expectation and variance of H, and

$$\begin{aligned}L &= 2H - t_a \\ \mathbb{E}[L] &= \mathbb{E}[2H - t_a] \\ &= 2 + t_a, \\ \mathbb{V}ar[L] &= \mathbb{V}ar[2H - t_a] \\ &= 4\mathbb{V}ar[H] = 4,\end{aligned}$$

for the expectation and variance of L. Following the definition of these marginal moments, we calculate the covariance in the branch lengths at each locus, $\mathbb{C}ov(L_A, L_B)$, as:

$$\begin{aligned}\mathbb{C}ov(L_A, L_B) &= \mathbb{E}[L_A L_B] - \mathbb{E}[L_A]\mathbb{E}[L_B] \\ \mathbb{E}[L_A L_B] &= \mathbb{E}[(2H_A - t_a)(2H_B - t_a)] \\ &= \mathbb{E}\left[4H_A H_B - 2t_a H_A - 2t_a H_B + t_a^2\right] \\ &= 4\mathbb{E}[H_A H_B] - 4t_a\mathbb{E}[H_A] + t_a^2 \\ &= 4(\mathbb{E}[T_A T_B] + 2t_a + t_a^2) - 4t_a(1 + t_a) + t_a^2.\end{aligned}$$

These derivations show that we can compute $\mathbb{C}ov(L_A, L_B)$ under the time-staggered sampling model by computing $\mathbb{E}[T_A T_B]$.

We approach this using a "staggered" version of the Simonsen-Churchill Model as described in the main text (Simonsen and Churchill 1997; Hobolth and Jensen 2014) (Figure A1). In the phase where $t < t_a$, with a single modern haplotype, we consider this as a two-state continuous-time Markov process with the rate matrix:

$$Q = \begin{bmatrix} -\frac{\rho}{2} & \frac{\rho}{2} \\ 1 & -1 \end{bmatrix},$$

which we use to solve for the probability that the ancestral process is in state x at time t_a as:

$$\begin{aligned}\mathbb{P}_{t_a}(x = (1,1,1)) &= \left(e^{\mathbf{Q}t_a}\right)_{0,1} \\ &= \frac{\rho(1 - e^{-t_a(\frac{\rho}{2}+1)})}{\rho + 2} \\ \mathbb{P}_{t_a}(x = (2,0,0)) &= 1 - \mathbb{P}_{t_a}(x = (1,1,1)),\end{aligned}$$

where the state $x = (2,0,0)$ represents 2 lineages that are ancestral to both locus A and locus B and the state $x = (1,1,1)$ represents 1 lineage ancestral to both locus A and B, one lineage ancestral to locus A, and one lineage ancestral to locus B (Hobolth and Jensen 2014; Simonsen and Churchill 1997). This corresponds to our "uncoupled state" in the main text. The two states in the Markov process with a single present haplotype can only be "coupled" ($(2,0,0)$) or "uncoupled" ($(1,1,1)$).

Returning to our computation of $\mathbb{E}[T_A T_B]$ in the second phase of the ancestral process ($t > t_a$), we obtain:

$$\begin{aligned}\mathbb{E}_{(2,0,0)}[T_A T_B] &= \frac{\rho^2 + 14\rho + 36}{\rho^2 + 13\rho + 18}, \\ \mathbb{E}_{(1,1,1)}[T_A T_B] &= \frac{\rho^2 + 13\rho + 24}{\rho^2 + 13\rho + 18}, \\ \mathbb{E}[T_A T_B] &= \mathbb{P}_{t_a}(x = (2,0,0))\mathbb{E}_{(2,0,0)}[T_A T_B] \\ &\quad + \mathbb{P}_{t_a}(x = (1,1,1))\mathbb{E}_{(1,1,1)}[T_A T_B] \\ &= \left(1 - \frac{\rho(1 - e^{-t(\frac{\rho}{2}+1)})}{\rho + 2}\right)\frac{\rho^2 + 14\rho + 36}{\rho^2 + 13\rho + 18} \\ &\quad + \frac{\rho(1 - e^{-t(\frac{\rho}{2}+1)})}{\rho + 2}\frac{\rho^2 + 13\rho + 24}{\rho^2 + 13\rho + 18},\end{aligned} \tag{8}$$

where $\mathbb{E}_x$ indicates the expectation conditional on starting in state x of the ancestral process. The first two expressions above are derived in Durrett 2008, Chapter 3, where both haplotypes are sampled at present. The last expression is a weighting of the expectations from different starting states in the two-locus ancestral process, where the weight corresponds to the probabilities that the modern haplotype is uncoupled at the time the ancient haplotype is sampled, t_a. From this we can compute the covariance in the branch

length, $\mathbb{C}ov(L_A, L_B)$ and $\mathbb{C}orr(L_A, L_B)$: by substituting the Equation 8 into the relevant expressions previously defined, leading to the expression:

$$\begin{aligned} \mathbb{C}orr(L_A, L_B) &= \frac{\mathbb{C}ov(L_A, L_B)}{\sqrt{Var(L_A)Var(L_B)}} \\ &= \mathbb{E}[T_A T_B] - 1, \end{aligned} \tag{9}$$

which simplifies to Equation 4 in the main text. The lower and upper limits of t_a are 0 and ∞, and we show the asymptotic behavior of $Corr(L_A, L_B)$ in terms of ρ:

$$\begin{aligned} \frac{2}{\rho+2} &< \mathbb{P}(x = (2,0,0)) \leq 1, \forall t_a \in [0, \infty) \\ Corr(L_A, L_B) &= \mathbb{E}[T_A T_B] - 1 \\ Corr(L_A, L_B)|t_a \to 0 &= \frac{\rho^2 + 14\rho + 36}{\rho^2 + 13\rho + 18} - 1 \\ &= \frac{\rho + 18}{\rho^2 + 13\rho + 18} \\ Corr(L_A, L_B)|t_a \to \infty &= \frac{2}{\rho+2}\frac{\rho^2 + 14\rho + 36}{\rho^2 + 13\rho + 18} \\ &\quad + \frac{\rho}{\rho+2}\frac{\rho^2 + 13\rho + 24}{\rho^2 + 13\rho + 18} - 1 \\ &= \frac{8\rho + 36}{\rho^3 + 15\rho^2 + 44\rho + 36}. \end{aligned}$$

This derivation highlights the change in the rate of decay in the correlation of the branch length as a function of the sampling time from $\mathcal{O}(\rho^{-1})$ to $\mathcal{O}(\rho^{-2})$.

To relate the correlation in total branch length to the correlation in the number of pairwise differences between two sequences, we use the following identities for the case where mutations occur as a Poisson process with rate $\theta/2$ along branches, where θ is the population-scaled mutation rate ($\theta = 4N_e\mu$) (Hobolth *et al.* 2019):

$$\begin{aligned} \pi_A|L_A &\sim \text{Pois}\left(\frac{\theta}{2}L_A\right), \\ \pi_B|L_B &\sim \text{Pois}\left(\frac{\theta}{2}L_B\right), \\ \mathbb{E}[\pi_A] &= \mathbb{E}[\pi_B] = \mathbb{E}[\mathbb{E}[\pi_A|L_A]] = \frac{\theta}{2}\mathbb{E}[L_A], \\ Var(\pi_A) &= \mathbb{E}[Var(\pi_A|L_A)] + Var(\mathbb{E}[\pi_A|L_A]), \\ &= \frac{\theta}{2}\mathbb{E}[L_A] + \left(\frac{\theta}{2}\right)^2 Var(L_A) \\ \mathbb{E}[\pi_A\pi_B] &= \mathbb{E}[\mathbb{E}[\pi_A\pi_B|L_A L_B]] = \frac{\theta^2}{4}\mathbb{E}[L_A L_B], \\ \mathbb{C}ov(\pi_A, \pi_B) &= \mathbb{E}[\pi_A\pi_B] - \mathbb{E}[\pi_A]\mathbb{E}[\pi_B] = \frac{\theta^2}{4}Cov(L_A, L_B), \\ \mathbb{C}orr(\pi_A, \pi_B) &= \frac{Cov(\pi_A, \pi_B)}{\sqrt{Var(\pi_A)Var(\pi_B)}}, \\ &= \frac{\frac{\theta^2}{4}Cov(L_A, L_B)}{\sqrt{\left(\frac{\theta}{2}\mathbb{E}[L_A] + \frac{\theta^2}{4}Var(L_A)\right)^2}} \\ &= \frac{\mathbb{C}ov(L_A, L_B)}{\frac{2}{\theta}\mathbb{E}[L_A] + Var(L_A)} \\ &= \frac{1}{1 + \frac{2+t_a}{2\theta}}(\mathbb{E}[T_A T_B] - 1), \end{aligned}$$

leading to a relationship with the correlation in the branch length at each locus, $\mathbb{C}orr(L_A, L_B)$:

$$\mathbb{C}orr(\pi_A, \pi_B) = \frac{1}{1 + \frac{2+t_a}{2\theta}}\mathbb{C}orr(L_A, L_B), \tag{10}$$

which is Equation 4 in the main text.

The Two-Locus Ancestral Process with population divergence and time-stratified sampling

In this section, we assume a model with divergence between the populations containing the ancient lineage and the modern lineage at the coalescent scaled time, t_{div}. We can partition the ancestral process into three phases: (1) when the modern lineage is the only one evolving, (2) when the ancient lineage and the modern lineage are both evolving *but are not able* to coalescent with one another and (3) when both lineages are in the ancestral population and can coalesce with each other. These three phases can be seen Figure A3.

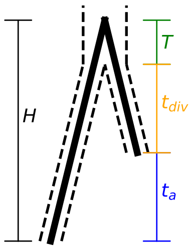

Figure A3 Description of variables in the single-locus case. H is the total tree height, T is the coalescent time of the ancient and modern lineage, and t_a is the sampling time of the ancient lineage (in coalescent units)

The model with population divergence has an additional parameter, t_{div}, the divergence time of the two populations. We first show the properties of the marginal tree under the divergence model (see Figure A3, for a definition of the quantities):

$$\begin{aligned}
\mathbb{E}[T] &= Var[T] = 1, \\
\mathbb{E}[H] &= \mathbb{E}[T + t_a + t_{div}] \\
&= 1 + t_a + t_{div}, \\
Var[H] &= Var[T + t_a + t_{div}] \\
&= 1, \\
\mathbb{E}[L] &= \mathbb{E}[2H - t_a] \\
&= 2\mathbb{E}[H] - t_a \\
&= 2(1 + t_a + t_{div}) - t_a \\
&= 2 + t_a + 2t_{div}, \\
Var[L] &= Var[2H - t_a] \\
&= 4Var[H] = 4,
\end{aligned}$$

where t_{div} is the population divergence times in coalescent units, t_a is the sampling time of the ancient lineage, T is the exponentially distributed time after both lineages are able to coalesce that they coalesce with one another. Using these results, we can calculate moments of the joint distribution of genealogical properties like the tree height (H), and total branch length (L). Specifically, the two-locus ancestral process behaves independently within each population for time t_a and t_{div} and each population is assumed to have the same population size. We begin by deriving the joint expectation of tree-height $H_A H_B$:

$$\begin{aligned}
\mathbb{E}[H_A H_B] &= \mathbb{E}[(T_A + t_a + t_{div})(T_B + t_a + t_{div})] \\
&= \mathbb{E}[T_A T_B] + 2t_{div} + 2t_a + (t_a + t_{div})^2,
\end{aligned}$$

and joint tree length $L_A L_B$:

$$\begin{aligned}
\mathbb{E}[L_A L_B] &= \mathbb{E}[(2H_A - t_a)(2H_B - t_a)] \\
&= 4\mathbb{E}[H_A H_B] - 4t_a + t_a^2,
\end{aligned}$$

where we must solve for the joint expectation of $\mathbb{E}[T_A T_B]$, but with the additional complication of population divergence. In order to do this we must calculate the probability of being in one of three starting states at time $t_a + t_{div}$: (1) the state $x = (2, 0, 0)$ where both the ancient and modern haplotypes are "coupled", (2) the state $x = (0, 2, 2)$ where both the ancient and modern haplotype are "uncoupled", which is possible due to the independent evolution of both lineages during $t_a < t < t_a + t_{div}$, and (3) state $x = (1, 1, 1)$ where one haplotype is uncoupled while the other is coupled. We consider the two independent processes within each population until the divergence time and calculate the probabilities of being in each starting state as follows:

$$\begin{aligned}\mathbb{P}(x=(2,0,0)|t_a,t_{div}) &= \mathbb{P}(x_1=(1,0,0)|t_a+t_{div})\mathbb{P}(x_2=(1,0,0)|t_{div}) \\ &= \frac{\rho e^{-(t_a+t_{div})(\rho/2+1)}+2}{\rho+2}\frac{\rho e^{-t_{div}(\rho/2+1)}+2}{\rho+2}, \\ \mathbb{P}(x=(0,2,2)|t_a,t_{div}) &= \mathbb{P}(x_1=(0,1,1)|t_a+t_{div})\mathbb{P}(x_2=(0,1,1)|t_{div}) \\ &= \frac{\rho(1-e^{-(t_a+t_{div})(\rho/2+1)})}{\rho+2}\frac{\rho(1-e^{-t_{div}(\rho/2+1)})}{\rho+2},\end{aligned}$$

and

$$\begin{aligned}\mathbb{P}(x=(1,1,1)|t_a,t_{div}) &= \mathbb{P}(x_1=(1,0,0)|t_a+t_{div})\mathbb{P}(x_2=(0,1,1)|t_{div}) \\ &\quad+\mathbb{P}(x_1=(0,1,1)|t_a+t_{div})\mathbb{P}(x_2=(1,0,0)|t_{div}) \\ &= \left(\frac{\rho e^{-(t_a+t_{div})(\rho/2+1)}+2}{\rho+2}\frac{\rho(1-e^{-t_{div}(\rho/2+1)})}{\rho+2}\right) \\ &\quad+\left(\frac{\rho(1-e^{-(t_a+t_{div})(\rho/2+1)})}{\rho+2}\frac{\rho e^{-t_{div}(\rho/2+1)}+2}{\rho+2}\right).\end{aligned}$$

From these probabilities we calculate the expectation of the joint coalescent times conditional on being in a specified state at time $t_a + t_{div}$ is obtained as:

$$\mathbb{E}[T_A T_B] = \sum_{x\in\{(1,1,1),(2,0,0),(0,2,2)\}} \mathbb{P}(x = x|t_a, t_{div})\mathbb{E}_x[T_A T_B],$$

where each of $\mathbb{E}_x[T_A T_B]$ are defined using previously derived results under the two-locus ancestral process conditional on being in a starting state x (Simonsen and Churchill 1997; Durrett 2008, Chapter 3). This is different from the model under population continuity (where the $x = (0,2,2)$ state was not possible). If we set $t_{div} = 0$, then this corresponds exactly to the model without population divergence. While the underlying mathematical results are more involved, they provide insights on how population divergence affects joint coalescent times.

We can now compute joint statistics (e.g. correlation) of the tree properties at each of the loci following common formulas, for example for the correlation in total branch length at each locus:

$$\mathbb{C}orr(L_A, L_B) = \mathbb{E}[T_A T_B] - 1.$$

Expectations of joint coalescent times under the time-stratified model

We assume that the following results on the joint coalescent times for two contemporary haplotypes starting in the same state in the two-locus ancestral process as defined in Durrett 2008, Chapter 3 are known:

$$\begin{aligned}\mathbb{E}_0[T_A T_B|x=(2,0,0)] &= \frac{\rho^2+14\rho+36}{\rho^2+13\rho+18} \\ \mathbb{E}_0[T_A T_B|x=(1,1,1)] &= \frac{\rho^2+13\rho+24}{\rho^2+13\rho+18} \\ \mathbb{E}_0[T_A T_B|x=(0,2,2)] &= \frac{\rho^2+13\rho+22}{\rho^2+13\rho+18},\end{aligned}$$

and now we will go through the individual cases for the time-stratified case: **(1)** both modern and ancient haplotypes start coupled, **(2)** both modern and ancient haplotypes are "uncoupled" and finally **(3)** where *only one* of the modern and ancient haplotypes are coupled (the other is uncoupled).

We first define two quantities, called γ and η. The variable γ refers to the probability of starting in the coupled $((1,0,0))$ state and ending in the uncoupled state $((0,1,1))$ at time t_a for a single haplotype (which is Equation (3) in the main text). The variable η is the converse, the probability of starting in the uncoupled state and ending in the coupled state at time t_a. Using the matrix exponential $e^{-\mathbf{Q}t_a}$ of the following rate matrix for the process with a single haplotype:

$$Q = \begin{bmatrix} -\frac{\rho}{2} & \frac{\rho}{2} \\ 1 & -1 \end{bmatrix},$$

we arrive at the following expressions for γ and η:

$$\begin{aligned}\gamma &= \frac{\rho(1-e^{-t_a(\frac{\rho}{2}+1)})}{\rho+2}, \\ \eta &= \frac{2(1-e^{-t_a(\frac{\rho}{2}+1)})}{\rho+2}.\end{aligned}$$

With these in hand we can start tackling our first case **(1)** from above:

$$\begin{aligned}\mathbb{E}_{t_a}[T_A T_B | x_{t_a} = (1,0,0), x_0 = (1,0,0)] = (1-\gamma)\mathbb{E}_0[T_A T_B | x = (2,0,0)] \\ + \gamma \mathbb{E}_0[T_A T_B | x = (1,1,1)],\end{aligned} \tag{11}$$

where, $x_0 = (1,0,0)$ indicates that the modern haplotype is coupled, and $x_{t_a} = (1,0,0)$ indicates that the ancient haplotype is coupled as well. This holds because the modern haplotype can be coupled with probability $1-\gamma$ leading to state $x = (2,0,0)$ for the joint ancestral process, or it can be uncoupled with probability γ resulting in state $x = (1,1,1)$. For case **(2)** (both haplotypes uncoupled) we obtain:

$$\begin{aligned}\mathbb{E}_{t_a}[T_A T_B | x_{t_a} = (0,1,1), x_0 = (0,1,1)] = (1-\eta)\mathbb{E}_0[T_A T_B | x = (0,2,2)] \\ + \eta \mathbb{E}_0[T_A T_B | x = (1,1,1)].\end{aligned} \tag{12}$$

The final case **(3)** is the most complicated and we break this into a further two sub-cases below:

$$\begin{aligned}\mathbb{E}_{t_a}[T_A T_B | x_{t_a} = (1,0,0), x_0 = (0,1,1)] = (1-\eta)\mathbb{E}_0[T_A T_B | x = (1,1,1)] \\ + \eta \mathbb{E}_0[T_A T_B | x = (2,0,0)], \\ \mathbb{E}_{t_a}[T_A T_B | x_{t_a} = (0,1,1), x_0 = (1,0,0)] = (1-\gamma)\mathbb{E}_0[T_A T_B | x = (1,1,1)] \\ + \gamma \mathbb{E}_0[T_A T_B | x = (0,2,2)],\end{aligned} \tag{13}$$

where the first case corresponds to the modern haplotype starting in the "uncoupled" state (denoted by the x_0 in the expectation) and the second case corresponds to the modern haplotype starting in the "coupled" state.

Appendix 2: The expected product of linkage disequilibrium between time-stratified samples

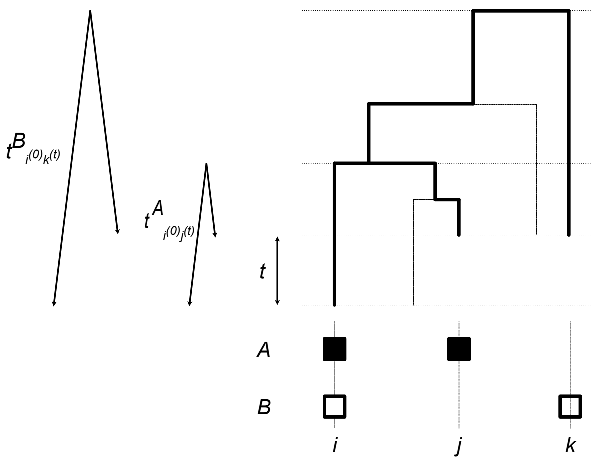

Figure A4 Schematic describing properties of lineages required for estimation of $\mathbb{E}[t^A_{i^{(0)}j^{(t)}} t^B_{i^{(0)}k^{(t)}}]$ in the case with time-stratification. Figure adapted from McVean (2002) for our case of time-stratification.

Here, we derive the scaled product of linkage disequilibrium between time-stratified samples normalized by the heterozygosity across both sites and time points. We first start from the definition of the statistic in terms of haplotype and allele frequencies in the ancient and modern samples:

$$\begin{aligned}\sigma_d^2 &= \frac{\mathbb{E}[D^{(0)} D^{(t)}]}{\mathbb{E}\left[p_A^0 (1-p_A^{(t)}) p_B^{(0)} (1-p_B^{(t)})\right]} \\ &= \frac{\mathbb{E}\left[(p_{AB}^{(0)} - p_A^{(0)} p_B^{(0)})(p_{AB}^{(t)} - p_A^{(t)} p_B^{(t)})\right]}{\mathbb{E}\left[p_A^{(0)} (1-p_A^{(t)}) p_B^{(0)} (1-p_B^{(t)})\right]},\end{aligned} \tag{14}$$

where $p_{AB}^{(t)}$ is the frequency of the haplotype with the derived alleles at both loci at time t, $p_A^{(t)}$ is the frequency of the derived allele at the first locus, and $p_B^{(t)}$ is the frequency of the derived allele at the second locus. Using the approach of McVean (2002) we define

this ratio using branch lengths in the genealogy relating modern and ancient samples, where a mutation would result in a observed pattern of identity by state (Figure A4). We first expand the numerator as follows:

$$\begin{aligned}\mathbb{E}[D^{(0)}D^{(t)}] &= \mathbb{E}[(p^0_{AB} - p^0_A p^0_B)(p^t_{AB} - p^t_A p^t_B)] \\ &= \mathbb{E}[p^{(0)}_{AB} p^{(t)}_{AB}] - \mathbb{E}[p^{(0)}_{AB} p^{(t)}_A p^{(t)}_B] - \mathbb{E}[p^{(0)}_A p^{(0)}_B p^{(t)}_{AB}] + \mathbb{E}[p^{(0)}_A p^{(0)}_B p^{(t)}_A p^{(t)}_B], \\ &\approx \frac{\mathbb{E}[I^A_{i^{(0)}j^{(t)}} I^B_{i^{(0)}j^{(t)}}] - \mathbb{E}[I^A_{i^{(0)}j^{(t)}} I^B_{i^{(0)}k^{(t)}}] - \mathbb{E}[I^A_{i^{(0)}j^{(t)}} I^B_{k^{(0)}j^{(t)}}] + \mathbb{E}[I^A_{i^{(0)}j^{(t)}} I^B_{k^{(0)}l^{(t)}}]}{\mathbb{E}[L^A L^B]},\end{aligned}$$

where i, j, k, l denote sampled haplotypes. Furthermore, $I^x_{(i^{(0)}j^{(t)})}$ is the branch length leading from the T_{mrca} of the samples $i^{(0)}$ at time 0 and $j^{(t)}$ at time t to the T_{mrca} of the total population (including the ancient individuals) at locus x. $\mathbb{E}[L^A L^B]$ is the joint expectation of the total genealogical branch length for the complete population at both loci. The approximation in the final step above follows from assuming a small mutation rate (McVean 2002). We use the definition $I^A_{i^{(0)}j^{(t)}} = T^A - t^A_{i^{(0)}j^{(t)}}$, where T^A is the T_{mrca} for the total population (modern and ancient) at locus A and $t^A_{i^{(0)}j^{(t)}}$ is the pairwise coalescent time for samples $i^{(0)}, j^{(t)}$ at locus A. Using this relationship between coalescent times and identity coefficients, we arrive at:

$$\begin{aligned}\mathbb{E}[D^{(0)}D^{(t)}] &= \mathbb{E}[(T^A - t^A_{i^{(0)}j^{(t)}})(T^B - t^B_{i^{(0)}j^{(t)}})] - \mathbb{E}[(T^A - t^A_{i^{(0)}j^{(t)}})(T^B - t^B_{i^{(0)}k^{(t)}})] \\ &\quad - \mathbb{E}[(T^A - t^A_{(i^{(0)}j^{(t)})})(T^B - t^B_{(k^{(0)}j^{(t)})})] + \mathbb{E}[(T^A - t^A_{i^{(0)}j^{(t)}})(T^B - t^B_{k^{(0)}l^{(t)}})] \\ &= \frac{\mathbb{E}[t^A_{i^{(0)}j^{(t)}} t^B_{i^{(0)}j^{(t)}}] - \mathbb{E}[t^A_{i^{(0)}j^{(t)}} t^B_{i^{(0)}k^{(t)}}] - \mathbb{E}[t^A_{i^{(0)}k^{(t)}} t^B_{i^{(0)}j^{(t)}}] + \mathbb{E}[t^A_{i^{(0)}j^{(t)}} t^B_{k^{(0)}l^{(t)}}]}{\mathbb{E}[L^A L^B]},\end{aligned}$$

where the product of pairwise coalescent times at one locus and the total T_{mrca} at the other locus (e.g. $\mathbb{E}[T^1 t^2_{(i^{(0)}j^{(0)})}]$) do not depend on the indices i, j (Durrett 2008, Chapter 3). This means that the numerator of the expression above can be computed using the expectations of pairwise coalescent times in the time-stratified model.

The denominator of our expression ($\mathbb{E}[p^{(0)}_A(1 - p^{(t)}_A)p^{(0)}_B(1 - p^{(t)}_B)]$) is the probability of drawing two haplotypes at the first locus that are at different time points and differ in their allelic identity, *and* drawing two haplotypes at the second locus from different timepoints that also differ in their allelic identity. This is a measure of the time-stratified joint heterozygosity at both sites. We note that this is different from the interpretation of $\mathbb{E}[p(1-p)q(1-q)]$ which is the probability of a difference at the first locus and a difference at the second locus under a random draw from of a sample from a contemporary population and is the denominator of σ^2_d (McVean 2002). We define the denominator similarly using pairwise coalescent times as:

$$\mathbb{E}[p^{(0)}_A(1 - p^{(t)}_A)p^{(0)}_B(1 - p^{(t)}_B)] \approx \frac{\mathbb{E}[t^A_{i^{(0)}j^{(t)}} t^B_{k^{(0)}l^{(t)})}]}{\mathbb{E}[L^A L^B]},$$

where we see that joint total branch length term $\mathbb{E}[L^A L^B]$ will cancel out when evaluating the ratio. We can now turn to actually computing this expression using the joint expectations for coalescent times calculated in our time-stratified model (see Appendix 1 for the derivation of these joint coalescent times):

$$\begin{aligned}\frac{\mathbb{E}[D^{(0)}D^{(t)}]}{\mathbb{E}[p^{(0)}_A(1 - p^{(t)}_A)p^{(0)}_B(1 - p^{(t)}_B)]} = \frac{1}{\mathbb{E}[T_A T_B | x_{t_a} = (0,1,1), x_0 = (0,1,1)]}\Big[&\mathbb{E}[T_A T_B | x_{t_a} = (1,0,0), x_0 = (1,0,0)] \\ &- \mathbb{E}[T_A T_B | x_{t_a} = (0,1,1), x_0 = (1,0,0)] \\ &- \mathbb{E}[T_A T_B | x_{t_a} = (1,0,0), x_0 = (0,1,1)] \\ &+ \mathbb{E}[T_A T_B | x_{t_a} = (0,1,1), x_0 = (0,1,1)]\Big],\end{aligned}$$

which can be simplified to the following expression after substituting the proper expressions for the joint coalescent times derived in Appendix 1:

$$\frac{(\rho+2)(\rho+10)}{\rho^3 e^{\frac{t(\rho+2)}{2}} + 15\rho^2 e^{\frac{t(\rho+2)}{2}} + 48\rho e^{\frac{t(\rho+2)}{2}} + 48 e^{\frac{t(\rho+2)}{2}} - 4},$$

which is the expression reported in the main text (Equation 7). Importantly, we find that when $t = 0$, the expression simplifies to $\frac{\rho+10}{\rho^2+13\rho+22}$ which is the expression for σ^2_d in the case with two contemporary samples (McVean 2002).

Appendix 3: Expected-Time to First Coalescent for an Ancient Sample

Here we consider a single ancient haplotype sampled at a time t_a in the past and how it coalesces into the ancestral lineages of a reference panel of size K haplotypes sampled at the present. We define the random variable T^* as the additional time of a coalescent

event involving the ancient haplotype and a lineage ancestral to the modern reference panel after the time that the ancient haplotype is sampled (t_a). The expectation of this quantity can be written as:

$$\begin{aligned}\mathbb{E}_{t_a,K}[T^*] &= \mathbb{E}[\mathbb{E}[T^*|A_K(t_a)]] \\ &= \mathbb{E}\left[\mathbb{E}\left[\sum_{j=2}^{A_K(t_a)+1} \mathbb{P}(I_j) \sum_{i=A_K(t_a)+1}^{j} T_i\right] |A_K(t_a)\right],\end{aligned}$$

where $A_K(t_a)$ is the number of lineages ancestral to the modern reference panel at time t_a, $\mathbb{P}(I_j)$ is the probability that the jth coalescent event involves the ancient lineage, and T_i is the ith inter-coalescent time.

Starting at time t_a with n_t lineages, we calculate the probability that the jth coalescent event involves the ancient lineage as:

$$\begin{aligned}\mathbb{P}(I_j) &= \left(1 - \frac{\binom{j-1}{2}}{\binom{j}{2}}\right) \prod_{k=A_n(t_a)}^{j+1} \frac{\binom{k-1}{2}}{\binom{k}{2}} \\ &= \frac{2}{j} \prod_{k=A_n(t_a)}^{j+1} \left(1 - \frac{2}{k}\right).\end{aligned}$$

In a constant population size model, we have $\mathbb{E}[T_j] = \frac{2}{j(j-1)}$. Using this fact, the expected time until the first coalescence involving the ancient lineage (T^*) is:

$$\begin{aligned}\mathbb{E}[T^*|A_K(t_a)] &= \mathbb{E}\left[\sum_{j=2}^{A_K(t_a)+1} \mathbb{P}(I_j) \sum_{i=A_K(t_a)+1}^{j} T_i\right] \\ &= \sum_{j=A_K(t_a)+1}^{2} \left[\frac{2}{j} \prod_{k=A_K(t_a)+1}^{j+1} \left(1 - \frac{2}{k}\right) \sum_{i=A_K(t_a)+1}^{j} \frac{2}{i(i-1)}\right],\end{aligned}$$

and considering the summation over $A_K(t_a)$, we arrive at our final expression:

$$\begin{aligned}\mathbb{E}_{t_a,K}[T^*] &= \mathbb{E}[\mathbb{E}[T^*|A_K(t_a)]] \\ &= \sum_{a=K}^{1} \mathbb{P}(A_K(t_a) = a) \left[\sum_{j=a+1}^{2} \left[\frac{2}{j} \prod_{k=a+1}^{j+1} \left(1 - \frac{2}{k}\right) \sum_{i=a+1}^{j} \frac{2}{i(i-1)}\right]\right].\end{aligned} \tag{15}$$

The probability distribution $\mathbb{P}(A_K(t) = a)$ involves a number of alternating sums and leads rapidly to numerical error as the sample size gets large (see Equation 15 in Chen and Chen (2013)). To alleviate this issue, following Jewett and Rosenberg (2014) we approximate $\mathbb{P}(A_K(t) = a)$ as $\delta(A_K(t) = \mathbb{E}[A_K(t)])$. That is, rather than calculate the probability distribution of $A_K(t)$ across states $1...K$, we will approximate it with its expectation $\mathbb{E}[A_K(t)]$. One approximation for $\mathbb{E}[A_K(t)]$ is found in Griffiths (1984):

$$\mathbb{E}[A_K(t)] \approx \frac{K}{K + (1-K)e^{-t}}.$$

Further approximations for this expectation exist and are explored in greater detail in Jewett and Rosenberg (2014). We chose the above approximation largely for computational convenience as it does not involve any summation, has a simple form, and is comparably accurate when compared to other approximations (Jewett and Rosenberg 2014).

The additional time to coalescence for the ancient sample ($\mathbb{E}[T^*]$) is proportional to the number of recombination events that can affect the genealogical closest haplotype to the ancient sample that is in the modern panel. For example, for a sample with $t_a = 2 \times 10^{-4}$ there is $\mathbb{E}[T^*] \approx 2 \times 10^{-3}$ and 2×10^{-4} with a panel size of $K = 1000$ and 10000 respectively (Figure S5). This guides the intuition that for large panel sizes and recent sampling times, the time for the ancient haplotype to coalesce with the panel is quite small, and therefore we expect the haplotype copying rate to be fairly small (leading to longer shared blocks). This is the key intuition behind long-range phasing methods that take advantage of recent relatedness (e.g. Loh *et al.* 2016). For samples on the order of $\sim 10^{-2}$ coalescent units, the relative ratio is 1.17 for $\mathbb{E}[T^*]$ with modern panel sizes of $K = 1000$ and $K = 10000$ (as opposed to 6.99 when $t_a = 10^{-4}$). This highlights a saturation effect of within-panel coalescence at deeper times, limiting the expected utility of large modern panels for the setting with substantially ancient samples (Figure S5).

Supplementary Figures

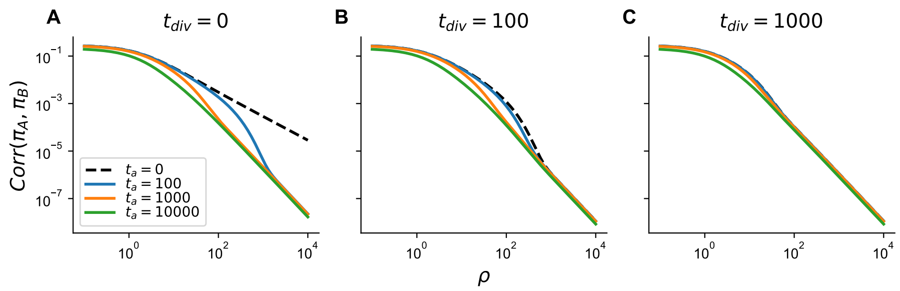

Figure S1 Theoretical correlation in pairwise differences for samples under a model with divergence. Divergence times t_{div} are **(A)** 0, **(B)** 100, and **(C)** 1000 generations in the past.

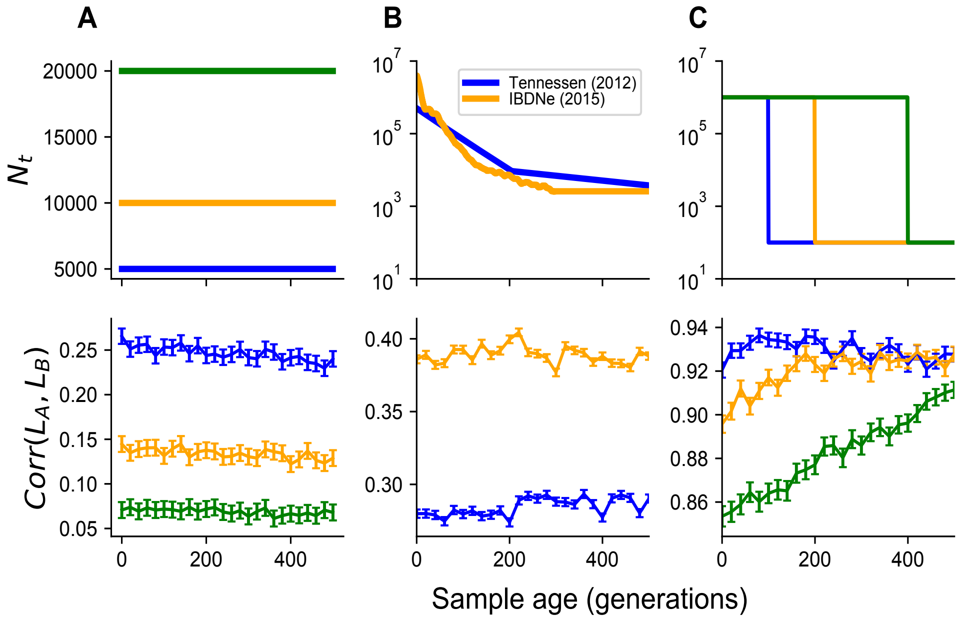

Figure S2 Effect of demographic history on the correlation in the total branch length between two loci. For all simulations, the recombination rate was set to 10^{-4} per generation. Simulated scenarios include: **(A)** constant population size, **(B)** inferred models of population growth, and **(C)** models of instantaneous population growth.

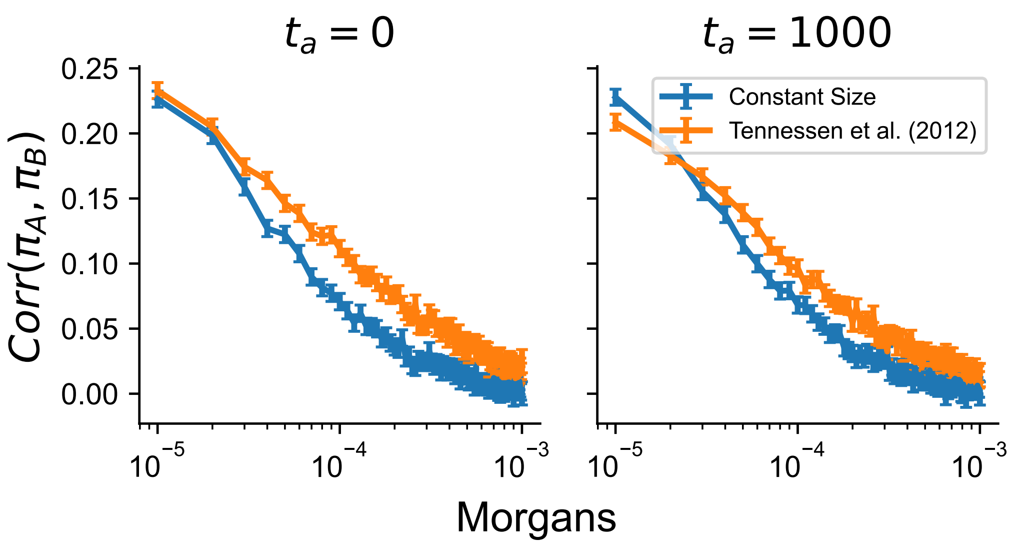

Figure S3 Effect of demographic history on the correlation in pairwise differences. Recombination points on the x-axis are considered as the mean recombination distance for windows of 1 kilobase separated by d windows (using $r = 10^{-8}$ as the recombination distance).

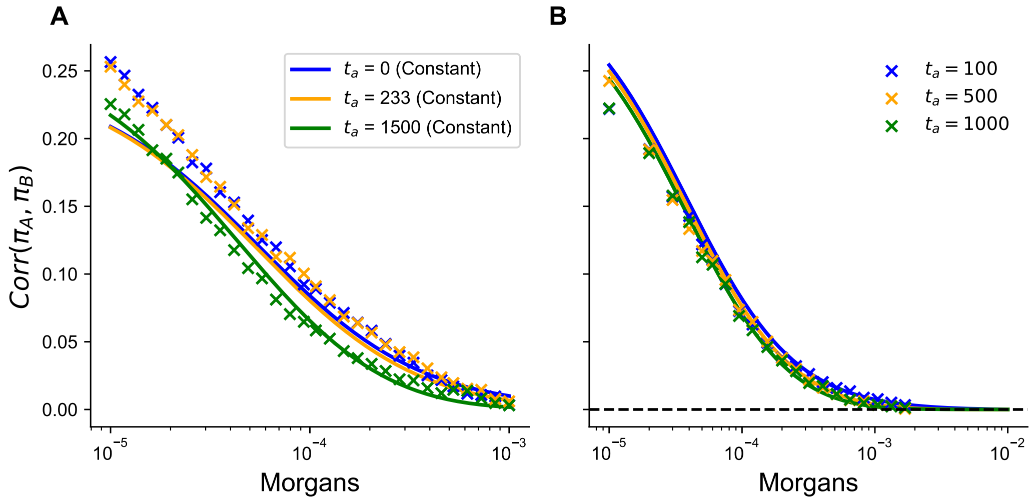

Figure S4 **A.** Effect of demographic history on the correlation in pairwise differences for time points related to *LBK* and *Ust-Ishim* samples under the model of (Tennessen *et al.* 2012) (crosses), compared against constant-sized theory (lines) for the same ages. **B** Comparison of constant-sized simulations at different time-depths against theoretical predictions from Equations (4).

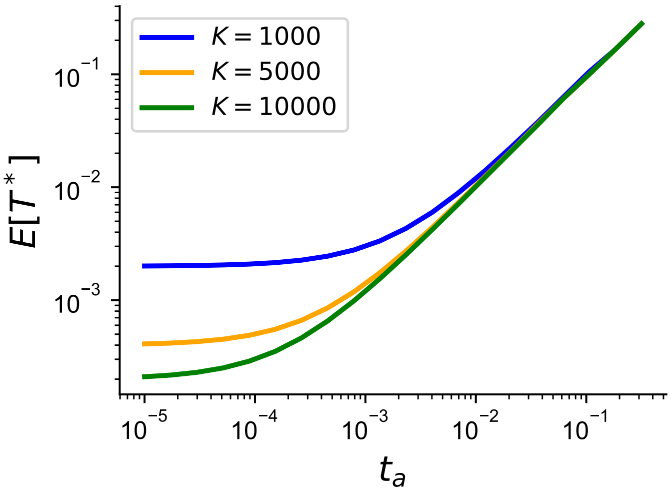

Figure S5 Expected time to first coalescent event involving an ancient haplotype with lineages ancestral to modern panel in a model of constant population size. As an approximation, with an $N_e = 10^4$, a sample with $t_a = 10^{-3}$ is 10 generations old($\sim$ 300 years old (Fenner 2005)), and there is little difference between reference panel sizes of $K = 5000$ and $K = 10000$.

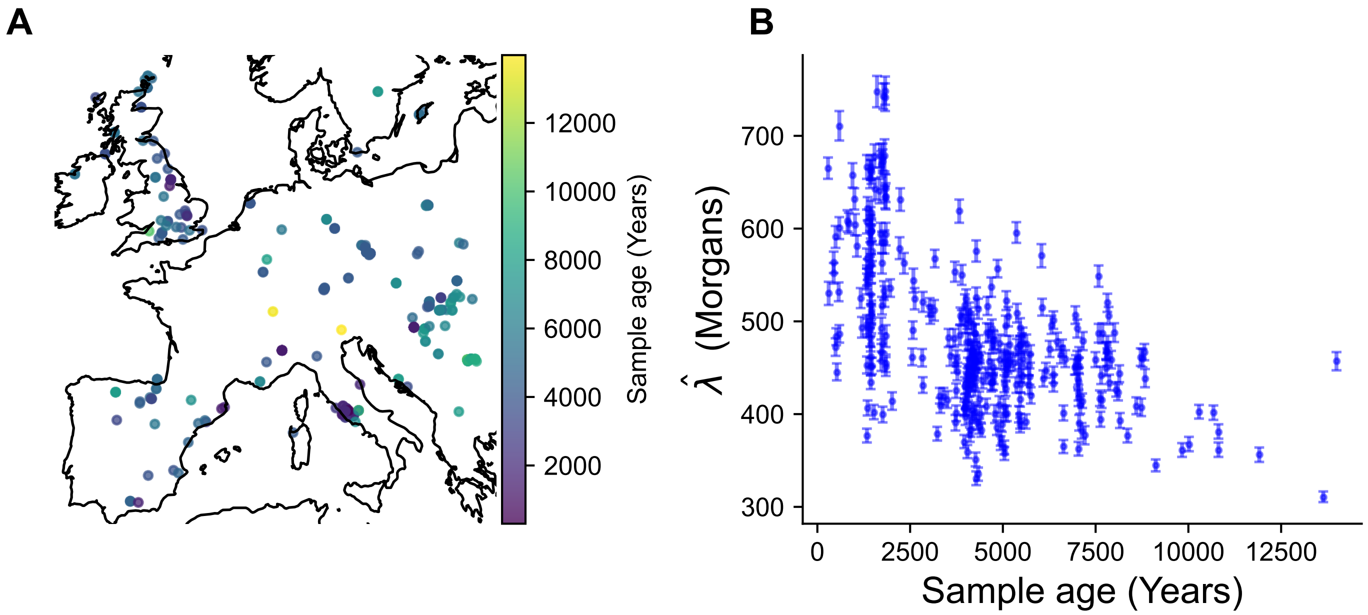

Figure S6 **(A)** Map of samples < 1500 kilometers from hypothetical location of central Europe (see Methods) **(B)** Decrease in estimated $\hat{\lambda}$ as a function of sample age in generations when estimated from male X chromosomes using all male X chromosomes from samples in the `CEU` population ($n = 49$) from Auton *et al.* (2015).

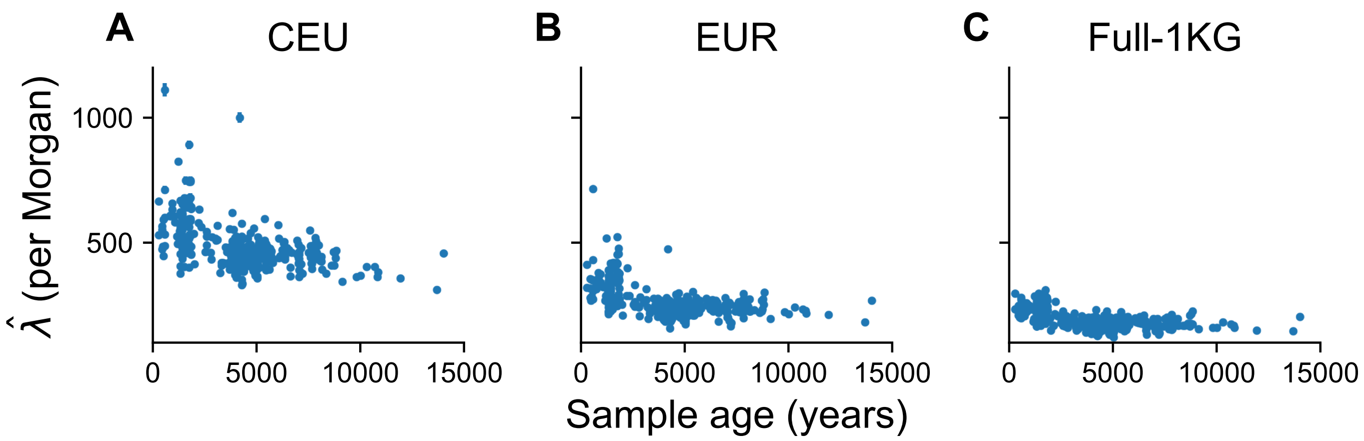

Figure S7 The impact of panel size on the estimated haplotype copying jump rate. **(A)** Using all male CEU individuals (n=49), **(B)** using all male individuals from the european (EUR) regional grouping (n=240), and **(C)** using all male individuals in the 1000 Genomes (n=1233). As the panel size increases, the estimated jump rate decreases in terms of the absolute scale, indicating longer shared haplotypes due to closer relatedness. However, in all cases we still find that the jump rate decrease as a function of the sample age ($p < 0.05$; linear regression). For all simulations we use the same set of samples as shown in the main text (restricting to < 1500 kilometers from a centroid in central europe).

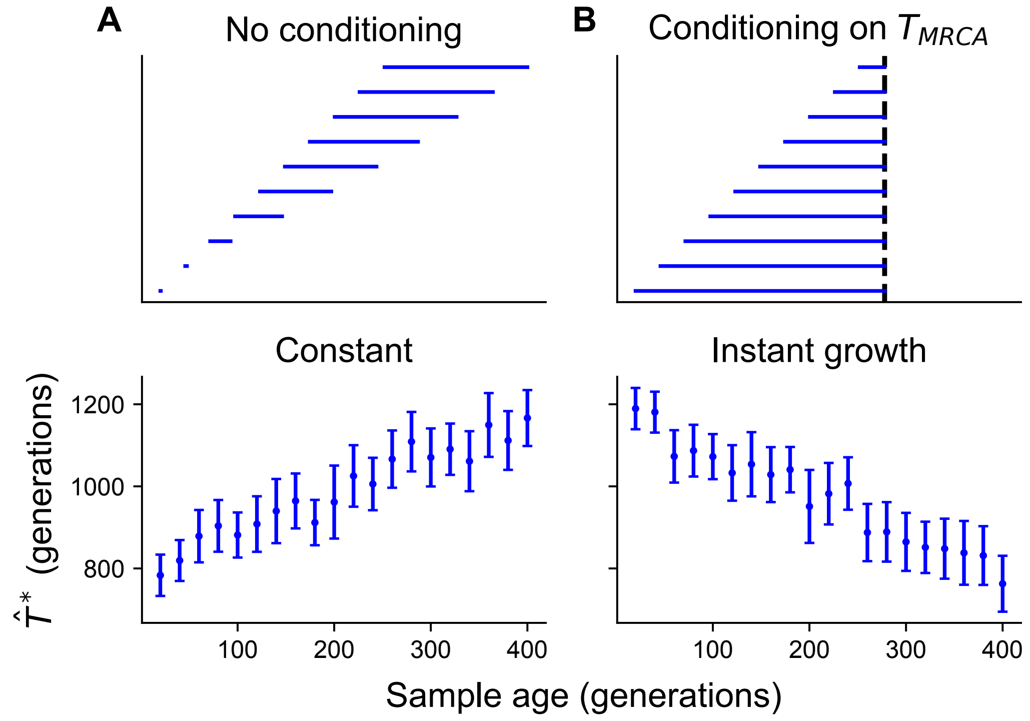

Figure S8 Schematic illustration of the impact of conditioning on the T_{mrca} with simulated data. **(A)** In the case of no-conditioning, we simulate the time to first coalescence for an ancient sample with a lineage ancestral to a member of a modern panel ($K = 100$) in a constant population size scenario of $N_e = 10^4$. **(B)** To mimic the case of conditioning on the T_{mrca}, we simulate instantaneous growth at 400 generations from a population size of 10^4 to 10^6. In both simulations, ancient haplotypes are sampled every 20 generations, are conducted using 5000 replicates and error bars represent 2 standard deviations from the mean time to first coalescence.

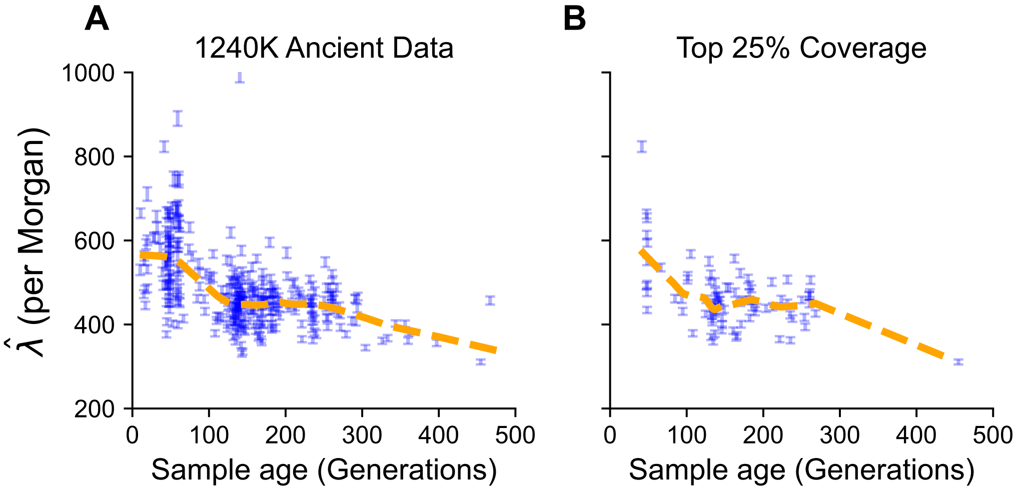

Figure S9 The effect of filtering by coverage on the estimated jump rate as a function of time. **(A)** The original dataset restricted to European individuals. **(B)** The evaluation of the haplotype copying jump rate restricted to individuals with the top 25% of the empirical autosomal coverage distribution. In both cases the qualitative decrease in the jump rate as a function of time is present.

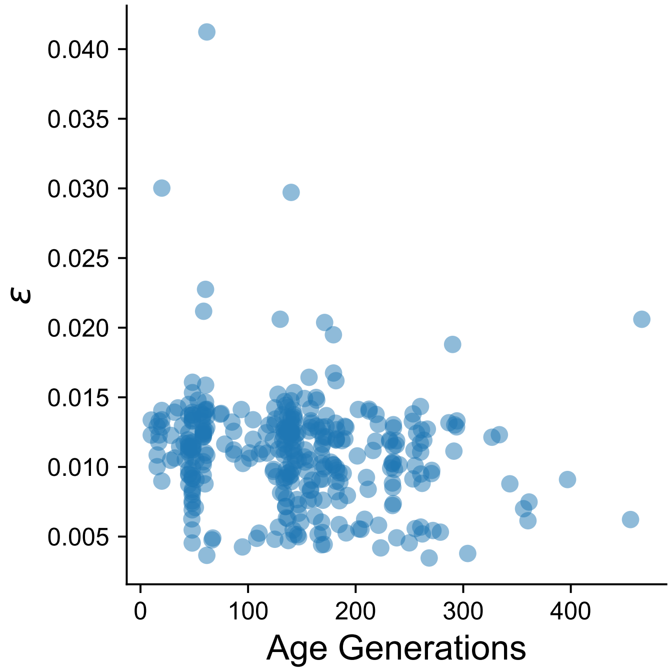

Figure S10 Estimated haplotype-copying error rate as a function of sample age. There is a weak anti-correlation between the estimated error rate and the sample age ($r = -0.17$, 95% CI=$[-0.27, -0.06]$).

Supplementary Data

**Sample Identifiers**	**Citation**
RISE479.SG, RISE98.SG	Allentoft et al. Population genomics of Bronze Age Eurasia. *Nature*. 2015 Jun 11;522(7555):167-72.
CL94, SZ18, CL92, SZ22, SZ7, SZ23, CL93, CL38, SZ36.SG, SZ3.SG, CL57, SZ5.SG, SZ2.SG, CL84, SZ16, SZ42, CL146, CL145, SZ27, CL121, SZ43.SG, SZ4.SG, SZ15.SG, CL30, SZ11.SG, SZ13, CL63, CL23, SZ45.SG	Amorim et al. Understanding 6th-century barbarian social organization and migration through paleogenomics. *Nature Communications*. 2018 Sep 11;9(1):3547.
R36.SG, R1286.SG, R53.SG, R68.SG, R1221.SG, R474.SG, R4.SG, R1287.SG, R47.SG, R115.SG, R70.SG, R108.SG, R1224.SG, R44.SG, R105.SG, R437.SG, R1219.SG, R59.SG, R969.SG, R111.SG, R31.SG, R63.SG, R64.SG, R136.SG, R33.SG, R1283.SG, R58.SG, R1545.SG, R132.SG, R107.SG, R15.SG, R61.SG, R55.SG, R7.SG, R30.SG, R130.SG, R116.SG, R34.SG, R113.SG, R851.SG, R1547.SG, R835.SG, R1551.SG, R104.SG, R52.SG, R1550.SG, R27.SG, R1548.SG, R970.SG, R1014.SG, R123.SG, R11.SG, R32.SG, R117.SG, R128.SG, R1543.SG, R131.SG, R1549.SG, R1220.SG, R850.SG, R1285.SG, R435.SG, R76.SG, R110.SG, R9.SG, R6.SG, R118.SG	Antonio et al. Ancient Rome: A genetic crossroads of Europe and the Mediterranean. *Science.* 2019 Nov 8;366(6466):708-714.
I6757, I6753_all.SG, I6767_all.SG, I6760_all.SG, I6747_all.SG, I6762_all.SG	Brace et al. Ancient genomes indicate population replacement in Early Neolithic Britain. *Nature Ecology and Evolution.* 2019 May;3(5):765-771.
rath1.SG, rath3.SG, rath2.SG	Cassidy et al. Neolithic and Bronze Age migration to Ireland and establishment of the insular Atlantic genome. *Proceedings of the National Academy of Sciences* 2016 Jan 12;113(2):368-73.
N47.SG, N17.SG, N27.SG, N20.SG, N49.SG, N26.SG, N28.SG	Fernandes et al. A genomic Neolithic time transect of hunter-farmer admixture in central Poland.*Scientific Reports.* 2018 Oct 5;8(1):14879.
Villabruna	Fu et al. The genetic history of Ice Age Europe. *Nature.* 2016 Jun 9;534(7606):200-5.
KO1_published.SG, NE5.SG, NE7.SG, NE6.SG, BR2.SG, IR1.SG	Gamba et al. Genome flux and stasis in a five millennium transect of European prehistory. *Nature Communications.* 2014 Oct 21;5:5257. doi: 10.1038/ncomms6257.
OC.SG, M95.SG, M96.SG	Gonzalez-Fortes et al. Paleogenomic Evidence for Multi-generational Mixing between Neolithic Farmers and Mesolithic Hunter-Gatherers in the Lower Danube Basin. *Current Biology.* 2017 Jun 19;27(12):1801-1810.e10.
Bichon.SG	Jones et al. Upper Palaeolithic genomes reveal deep roots of modern Eurasians. *Nature Communications.* 2015 Nov 16;6:8912.

I2793_published, I2753, I1904, I2367_published, I0449, I5838, I2369, I3269, I2377, I1565, I2379, I1878_published, I2739_published, I2366_published, I1877_all, I2384_published, I2783, I1880, I3276_published, I2788, I2791_published, I0821_published, I0659_published	Lipson et al. Parallel palaeogenomic transects reveal complex genetic history of early European farmers. *Nature.* 2017 Nov 16;551(7680):368-372.
3DT26.SG, 6DT23.SG, 6DT21.SG, 3DT16.SG, 6DT3.SG, 6DT22.SG, 6DT18.SG, NO3423.SG, I0099_published, I0114, I0012, I0172, I0104, I0017, I0412, I0406, I0585, I1504, I1495, I1507, I1496, I1500	Martiniano et al. Genomic signals of migration and continuity in Britain before the Anglo-Saxons. *Nature Communications.* 2016 Jan 19;7:10326.
I4882, I4089, I4870, I5070, I5240, I5204, I4916, I4331, I5207, I5235, I5402, I4914_published, I5237, I5077, I4881_published, I4878, I1131, I4915, I5078, I3947, I5232, I5411, I3948, I0676, I0634, I5236, I4880	Mathieson et al. The genomic history of southeastern Europe. *Nature.* 2018 Mar 8;555(7695):197-203.
POST_6, WEHR_1192SkA, OBKR_80, AITI_43, UNTA85_1343	Mittnik et al. Kinship-based social inequality in Bronze Age Europe. *Science.* 2019 Nov 8;366(6466):731-734.
I7207, I6696, I6677, I7208, I7949, I7209	Narasimhan et al. The formation of human populations in South and Central Asia. *Science.* 2019 Sep 6;365(6457).
LaBrana1_published.SG	Olalde et al. Derived immune and ancestral pigmentation alleles in a 7,000-year-old Mesolithic European. *Nature.* 2014 Mar 13;507(7491):225-8.
I2787, I7279, I7572, I7286, I6774, I7043, I6581, I1382, I7249, I2478, I2606, I4304, I2660, I7287, I3875, I3135, I7042, I2452, I2445, I2860, I2932, I2630, I2935, I2447, I5835, I2859, I4893, I5514, I4889, I2933, I4888, I4886, I3134, I2978, I3132, I3133, I2979, I2618, I4887, I2977, I5748, I4884, I4895, I4891, I2631, I5377, I4070, I2602, I4303, I7276, I5833, I5118, I2417, I2650, I2597, I2635, I2365, I7289, I7282, I7269, I2786, I3256, I2364, I1767, I2567, I2741, I4073, I5379, I7280, I4885, I4069, I2691, I7044, I5750, I6759, I5513, I6680, I7272, I3041, I2655, I2457, I7275, I7554, I4074, I7640, I2637, I6531, I7212, I7210, I7251, I5519, I1381, I7278	Olalde et al. The Beaker phenomenon and the genomic transformation of northwest Europe. *Nature.* 2018 Mar 8;555(7695):190-196.
I7606, I8209, I11248, I12209, I7642, I3759, I8364, I7602, EHU002, I8569, I7604, I8199, I3756, I1840, I10287_published, I3494, I8365, I0843, I11249, I4565, I10895, I12410, I3983	Olalde et al. The genomic history of the Iberian Peninsula over the past 8000 years. *Science.* 2019 Mar 15;363(6432):1230-1234.
Loschbour_snpAD.DG	Prüfer et al. A high-coverage Neandertal genome from Vindija Cave in Croatia. *Science.* 2017 Nov 3;358(6363):655-658. doi: 10.1126/science.aao1887.
prs009-ALL_DATA.SG, prs013-ALL_DATA.SG, ans017.SG, prs016-ALL_DATA.SG, ans008.SG, ans014.SG	Sanchez-Quinto et al. Megalithic tombs in western and northern Neolithic Europe were linked to a kindred society. *Proceedings of the National Academy of Sciences* 2019 May 7;116(19):9469-9474.
I0156.SG, I0160.SG	Schiffels et al. Iron Age and Anglo-Saxon genomes from East England reveal British migration history. *Nature Communications*. 2016 Jan 19;7:10408.

RISE1162.SG, RISE1241.SG, RISE1173.SG, RISE1168.SG, RISE1160.SG, RISE1163.SG, RISE1169.SG, RISE1165.SG, RISE1171.SG	Schroeder et al. Unraveling ancestry, kinship, and violence in a Late Neolithic mass grave. *Proceedings of the National Academy of Sciences* 2019 May 28;116(22):10705-10710.
Ajvide58.SG	Skoglund et al. Genomic diversity and admixture differs for Stone-Age Scandinavian foragers and farmers. *Science.* 2014 May 16;344(6185):747-50.

Table S1 Sample identifier information and corresponding publication citation for ancient DNA samples used from Allen Ancient DNA Resource.
